# Antimicrobial and Antioxidant Polyketides from a Deep-Sea-Derived Fungus *Aspergillus versicolor* SH0105

**DOI:** 10.3390/md18120636

**Published:** 2020-12-11

**Authors:** Lu-Jia Yang, Xiao-Yue Peng, Ya-Hui Zhang, Zhi-Qing Liu, Xin Li, Yu-Cheng Gu, Chang-Lun Shao, Zhuang Han, Chang-Yun Wang

**Affiliations:** 1 Key Laboratory of Marine Drugs, The Ministry of Education of China, School of Medicine and Pharmacy, Institute of Evolution & Marine Biodiversity, Ocean University of China, Qingdao 266003, China; yanglujia@stu.ouc.edu.cn (L.-J.Y.); pengxiaoyue@stu.ouc.edu.cn (X.-Y.P.); zhangyahui@stu.ouc.edu.cn (Y.-H.Z.); liuzhiqing@ouc.edu.cn (Z.-Q.L.); lixin8962@ouc.edu.cn (X.L.); shaochanglun@ouc.edu.cn (C.-L.S.); 2 Laboratory for Marine Drugs and Bioproducts, Qingdao National Laboratory for Marine Science and Technology, Qingdao 266237, China; 3 Jealott’s Hill International Research Centre, Syngenta, Bracknell, Berkshire RG42 6EY, UK; yucheng.gu@syngenta.com; 4 Institute of Deep-sea Science and Engineering, Chinese Academy of Science, Sanya 572000, China

**Keywords:** *Aspergillus versicolor*, deep-sea-derived fungus, polyketide, antimicrobial activity, antioxidant activity

## Abstract

Fifteen polyketides, including four new compounds, isoversiol F (**1**), decumbenone D (**2**), palitantin B (**7**), and 1,3-di-*O*-methyl-norsolorinic acid (**8**), along with 11 known compounds (**3**–**6** and **9**–**15**), were isolated from the deep-sea-derived fungus *Aspergillus versicolor* SH0105. Their structures and absolute configurations were determined by comprehensive spectroscopic data, including 1D and 2D NMR, HRESIMS, and ECD calculations, and it is the first time to determine the absolute configuration of known decumbenone A (**6**). All of these compounds were evaluated for their antimicrobial activities against four human pathogenic microbes and five fouling bacterial strains. The results indicated that 3,7-dihydroxy-1,9-dimethyldibenzofuran (**14**) displayed obvious inhibitory activity against *Staphylococcus aureus* (ATCC 27154) with the MIC value of 13.7 μM. In addition, the antioxidant assays of the isolated compounds revealed that aspermutarubrol/violaceol-I (**15**) exhibited significant 1,1-diphenyl-2-picrylhydrazyl (DPPH) radical scavenging activity with the IC_50_ value of 34.1 μM, and displayed strong reduction of Fe^3+^ with the ferric reducing antioxidant power (FRAP) value of 9.0 mM under the concentration of 3.1 μg/mL, which were more potent than ascorbic acid.

## 1. Introduction

Marine-sourced microbes have been deemed as one of the important resources for the discovery of drug lead compounds, with increasing number of diverse new bioactive natural products reported in recent years [1]. A series of remarkable progress have been made in the exploitation of marine microbial resources using various technical strategies, for instance, epigenetic modification [2,3], coculture [4,5], and genome mining [6,7]. The genus *Aspergillus* was widely distributed in marine environment and marine-derived *Aspergillus* species was home to a crucial reservoir for producing new bioactive chemical molecules to promote the development of marine drugs [8,9]. So far, plenty of novel and active secondary metabolites have been reported from *Aspergillus*, such as anticancer plinabulin (NPI-2358) [10], *α*-glucosidase inhibitor aspergillusol A [11], and antiviral ochraceopone A and isoasteltoxin [12]. Inspiringly, plinabulin (NPI-2358) was an inhibitor of tubulin polymerization in third phase of clinical study to treat metastatic advanced nonsmall cell lung cancer (NSCLC) [13]. It was noteworthy that the studies of microbial secondary metabolites from extreme marine environments like deep sea have been gradually brought to the forefront in recent decades [14,15,16]. More and more new bioactive natural products have been discovered from the deep-sea derived *Aspergillus*, e.g., antifungal versicoloids A and B [17], cytotoxic penicillenols A1 and B1 [18], and anti-inflammatory cyclopenin [19].

**Figure 1:**
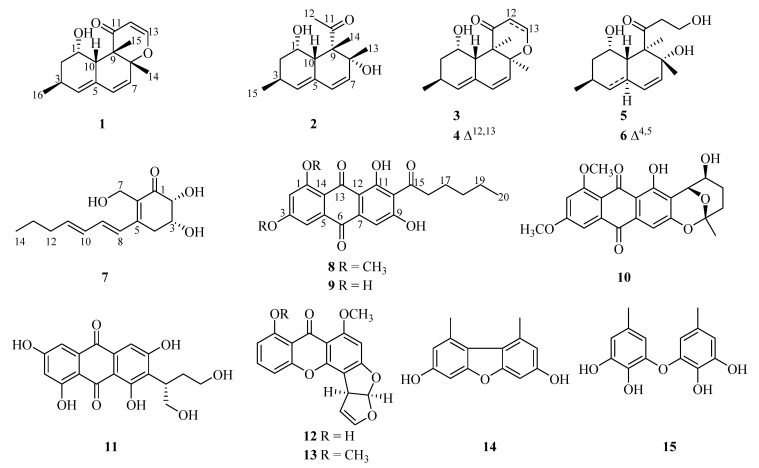
The structures of isolated compounds **1**–**15.**

As a part of our continuous research for marine bioactive natural products, a variety of bioactive compounds have been obtained from marine-derived *Aspergillus* genus [20], such as antibacterial (−)-sydonic acid [21], anti-RSV 22-*O*-(*N*-Me-*L*-valyl)-21-*epi*-aflaquinolone B [22], and antituberculous asperversiamides A–C [23]. Recently, a deep-sea-derived fungus *Aspergillus versicolor* SH0105 isolated from a Mariana Trench sediment sample (−5455 m) attracted our attention owing to its EtOAc extract of the fungal culture exhibiting antibacterial activity. The further chemical investigation on the EtOAc extract led to the isolation of four new polyketides (**1**–**2** and **7**–**8**), along with 11 known compounds (**3**–**6** and **9**–**15**) (Figure 1). Herein, we report the isolation, structure elucidation, and biological activities of these compounds.

## 2. Results and Discussion

**Figure 2:**
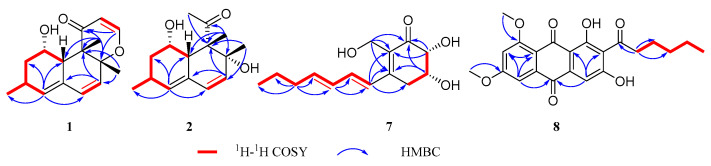
The key ^1^H-^1^H COSY and HMBC correlations of **1**–**2** and **7**–**8.**

Isoversiol F (**1**) was obtained as a yellowish oil with a molecular formula of C_16_H_20_O_3_ on the basis of the HRESIMS at *m*/*z* 261.1492 [M + H]^+^ (calcd for 261.1485) (Figure S9), displaying the same molecular formula with the coisolated 12,13-dedihydroversiol (**4**), which was first isolated from the marine-derived *Aspergillus* sp. SCS-KFD66 [24]. The ^1^H NMR, ^13^C NMR, and HSQC spectra of **1** (Table 1 and Figures S2–S4) revealed the presence of one carbonyl (*δ*_C_ 200.2), one olefinic quaternary carbon (*δ*_C_ 130.3), five olefinic methine, two sp^3^ quaternary carbons (with one oxygenated (*δ*_C_ 86.2)), one methylene (*δ*_C_ 38.8, *δ*_H_ 1.30 and 2.00), three methines (including one oxymethine *δ*_C_ 67.5, *δ*_H_ 5.20), and three methyls. These structural features were also very similar to those of **4**. The ^1^H-^1^H COSY interactions and the key HMBC correlations from H-4 to C-2, C-6, C-10, and C-16, from H-13 to C-8 and C-11, from H_3_-14 to C-7 and C-9, and from H_3_-15 to C-8, C-9, C-10, and C-11 in **1** demonstrated the same planar structure with **4** (Figure 2 and Figures S5 and S6). The obvious distinctions were the chemical shifts of the oxymethine (*δ*_C_ 67.5 and *δ*_H_ 5.20) and methyl (*δ*_C_ 17.2 and *δ*_H_ 1.28) in **1** replaced the oxymethine (*δ*_C_ 66.8 and *δ*_H_ 3.95) and methyl (*δ*_C_ 13.5 and *δ*_H_ 1.17) in **4**, respectively, which manifested that compound **1** should be a diastereoisomer of **4**.

**Figure 3:**
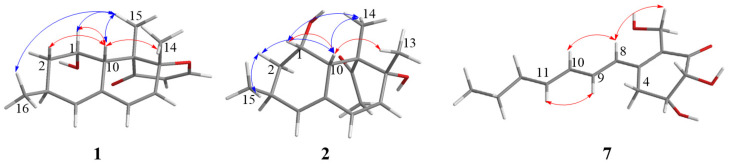
The NOE (blue) and NOESY (red) correlations of **1**–**2** and **7.**

**Figure 4:**
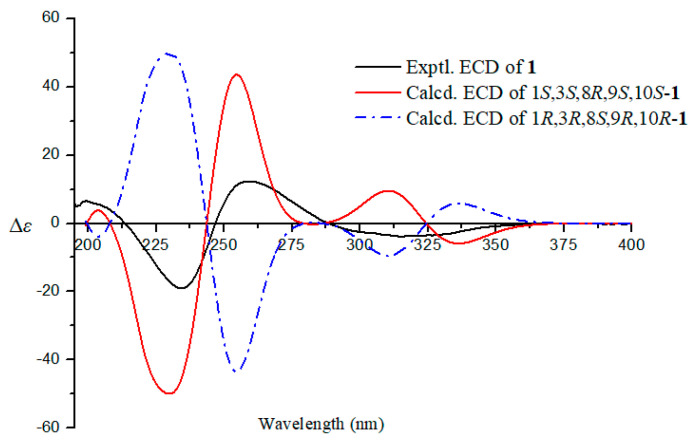
The experimental and calculated ECD spectra of **1.**

The relative configuration of **1** was determined by coupling constants, 1D NOE and 2D NOESY spectra. The small coupling constant of *J*_H-1, H-10_ = 3.3 Hz reflected the *syn*-relationship of H-1 and H-10. In the NOE spectrum, the irradiation of H_3_-15 (*δ*_H_ 1.28) led to the signal increase of H-1 (*δ*_H_ 5.20) and H_3_-16 (*δ*_H_ 1.04), suggesting that H_3_-15, H_3_-16, and H-1 should be positioned at the same planar (Figure 3 and Figure S8). Besides, in the NOESY spectrum, the correlation was also observed between H-10 and H_3_-14 indicating the same face of these protons (Figure 3 and Figure S7). Thus, the relative configuration of **1** was assumed as 1S^*^,3S^*^,8R^*^,9S^*^,10S^*^. The Mosher method was applied to determine the absolute configuration of **1**, however, it failed. Fortunately, the absolute configuration of **1** was resolved by ECD calculations. Its experimental ECD spectrum agreed with that of calculated 1*S*,3*S*,8*R*,9*S*,10*S*-**1**, which exhibited negative Cotton effect at around 230 nm and positive Cotton effect at around 260 nm (Figure 4 and Figure S32). Therefore, the absolute configuration of **1** was assigned as 1*S*,3*S*,8*R*,9*S*,10*S*. Compound **1** was a derivative of versiol [25], therefore, we named it as isoversiol F, which followed the reported isoversiols A–E [26].

Decumbenone D (**2**) was also obtained as a yellowish oil and assigned the molecular formula C_15_H_22_O_3_ by HRESIMS at *m/z* 233.1543 [M − H_2_O + H]^+^ (calcd for 233.1536) (Figure S18), with five degrees of unsaturation. The NMR data of **2** (Table 1 and Figures S10–S12) indicated the presence of one ketone carbonyl (*δ*_C_ 216.3), four olefinic signals (one quaternary), two sp^3^ quaternary carbons (with one oxygenated), three methines (including one oxymethine *δ*_C_ 67.3, *δ*_H_ 4.22), one methylene (*δ*_C_ 40.8, *δ*_H_ 1.85 and 1.22), and four methyl groups. These spectroscopic features suggested the presence of a similar skeleton with those of coisolated decumbenone A (**6**), which was first discovered from the fungus *Penicillium decumbens* [27]. The distinct differences were the existence of an additional methyl group (*δ*_C_ 30.8 and *δ*_H_ 2.28) in **2**, and the absence of two methylenes (including one oxygenated) of the side chain in **6**, indicating an acetyl group [CH_3_CO−] of the side chain at C-9 in **2** replaced the 3-hydroxypropionyl group [HOCH_2_CH_2_CO−] in **6**, which was verified by the HMBC correlations from H-12 to C-1 and C-9 (Figure 2 and Figure S14).

**Figure 5:**
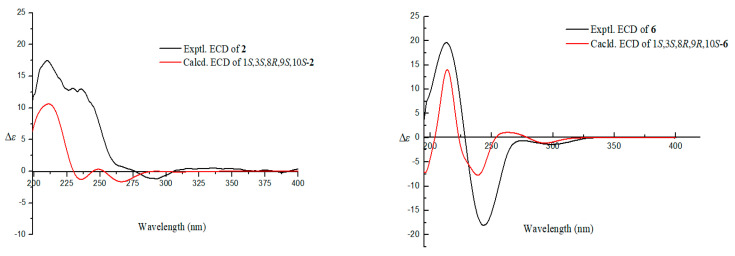
The experimental and calculated ECD spectra of **2** and **6.**

The relative configuration of **2** was also determined by coupling constants, 1D NOE, and 2D NOESY spectra. The small coupling constant of *J*_H-1, H-10_ = 3.3 Hz demonstrated the same side of H-1 and H-10. In the 1D NOE experiment measured in CDCl_3_ (Figure 3 and Figure S17), the irradiation of H_3_-14 (δ_H_ 1.46) enhanced the signal of H-10 (*δ*_H_ 2.98), and the irradiation of H-10 and H_3_-15 (*δ*_H_ 1.05) simultaneously resulted the enhancement of H-2b (δ_H_ 1.32), implying that H_3_-14, H_3_-15, and H-10 should be placed at the same face (Figure 3). In addition, the NOESY cross-peaks between H-10 and H_3_-13 indicated that these protons also should be coplanar (Figure 3 and Figure S15). Herein, the relative configuration of **2** was deduced as 1S^*^,3S^*^,8R^*^,9S^*^,10S^*^. The calculated ECD spectrum of 1*S*,3*S*,8*R*,9*S*,10*S*-**2** matched the experimental carve of **2** (Figure 5 and Figure S33). Therefore, the absolute configuration of **2** was assumed as1*S*,3*S*,8*R*,9*S*,10*S*. It was worth mentioning that only the relative stereochemistry of the known compound **6** was assumed by Fujii et al. [27]. Herein, we firstly determined the absolute configuration of **6** as 1*S*,3*S*,8*R*,9*R*,10*S* by comparing the experimental and calculated ECD spectra (Figure 5 and Figure S35).

Palitantin B (**7**) was isolated as a yellow solid. Its molecular formula was suggested to be C_14_H_20_O_4_ according to its HRESIMS at *m*/*z* 253.1442 [M + H]^+^ (calcd for 253.1434) (Figure S25), with five degrees of unsaturation. The ^1^H NMR, ^13^C NMR data of **7** (Table 1 and Figures S19 and S20) displayed the presence of one carbonyl group, six olefinic carbons, two oxymethines, four methylenes, and one methyl group. In the ^1^H-^1^H COSY spectrum, the cross-peaks of H-2/H-3/H-4 and correlations between H-8 to H-14 demonstrated a residue of [–OCHCH(O)CH_2_–] and an aliphatic spin system C-8 to C-14, respectively, which was also verified by the corresponding HMBC correlations (Figure 2 and Figures S22 and S23). The observed HMBC correlations from H-8 to C-4, C-5, and C-6 and from H-7 to C-1, C-5, and C-6 suggested the aliphatic chain should be located at C-5 and the hydroxymethyl linked at C-6 (Figure 2). Because of the demand of five degrees of unsaturation, an additional ring should be proposed, which was confirmed by the HMBC correlations from H-3 to C-1 and C-5, and H-2 to C-1 and C-6 (Figure 2). Hence, the planar structure of **7** was determined, which was similar with the known palitantin isolated from a plant endophytic *A. fumigatiaffinis* [28], except that the saturated bond at C-5/C-6 in palitantin was replaced by a double bond in **7**.

**Figure 6:**
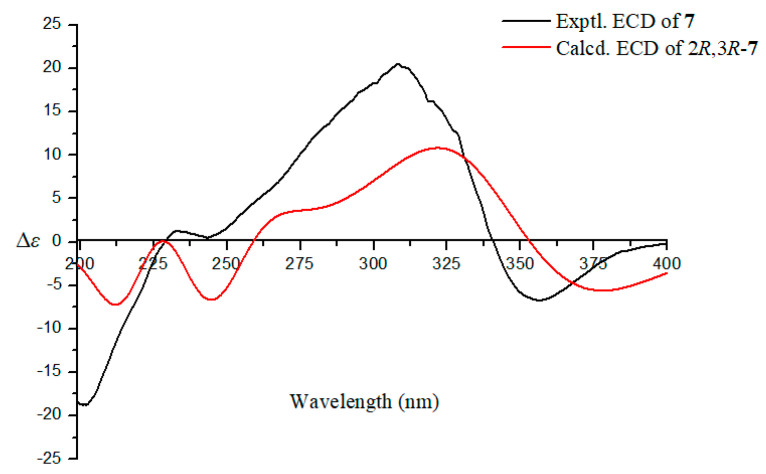
The experimental and calculated ECD spectra of **7.**

The relative configuration of **7** was determined by coupling constants and NOESY correlations. The coupling constant of *J*_H-2, H-3_ = 3.0 Hz suggested the *syn*-relationship of H-2 and H-3. The *E*-configuration of the two double bonds was elucidated by the large coupling constant *J*_H-10, H-11_ = 15.1 Hz and the NOESY correlations of H-9/H-11 and H-8/H-10 (Figure 3 and Figure S24). The absolute configuration of **7** was investigated by quantum chemical TDDFT calculations of its ECD spectrum. The experimental ECD spectrum was consistent with the calculated one of 2*R*,3*R*-**7** (Figure 6 and Figure S34), suggesting the absolute configuration of **7** as 2*R*,3*R*.

1,3-Di-*O*-methyl-norsolorinic acid (**8**) was isolated as a red powder and assigned the molecular formula as C_22_H_22_O_7_ based on its HRESIMS data (Figure S31), including 12 degrees of unsaturation. The ^1^H NMR spectrum (Table 2 and Figure S26) displayed one active hydrogen signal (*δ*_C_ 13.9), three aromatic protons (*δ*_C_ 6.99 (d, *J* = 2.5 Hz), 7.08 (s), 7.24 (d, *J* = 2.5 Hz)), two oxymethyl groups, four methylenes, and one methyl. The ^13^C NMR spectrum (Table 2 and Figure S27) showed the presence of 3 carbonyl groups and 12 aromatic carbons. These spectroscopic features were analogous to those of coisolated norsolorinic acid (**9**), which was obtained from the fungus *Emericella navahoensis* [29], except for two additional oxymethyl groups in **8**. The HMBC correlations from these two oxymethyl groups to C-1 and C-3 revealed that they were attached to C-1 and C-3, respectively (Figure 2 and Figure S30). Therefore, the structure of **8** was determined.

The structures of all known compounds, versiol (**3**) [25], 12,13-dedihydroversiol (**4**) [24], decumbenones B (**5**) and A (**6**) [27], norsolorinic acid (**9**) [29], 6,8-di-*O*-methylaverufin (**10**) [30], versiconol (**11**) [31], sterigmatocystin (**12**) [32], *O*-methylsterigmatocystin (**13**) [33], 3,7-dihydroxy-1,9-dimethyldibenzofuran (**14**) [34], aspermutarubrol/violaceol-I (**15**) [35,36], were elucidated by NMR, MS data and comparing with those of reported literature.

Antimicrobial resistance phenomenon is still a global issue, which is threatening the human’s life [37,38], indicating that it is very urgent to discover new antimicrobial molecules or mechanisms. In this study, all the isolated compounds **1**–**15** were evaluated for their antimicrobial activities against four human pathogenic microbes and five fouling bacterial strains. The results suggested that compound **14** displayed strong inhibitory activity against *Staphylococcus aureus* (ATCC 27154) with the MIC value of 13.7 μM, which was comparable to the positive control ciprofloxacin (MIC = 9.4 μM), and presented moderate inhibitory activity against *Aeromonas salmonicida* (ATCC 7965D) with the same MIC value of 13.7 μM (sea nine 211, MIC = 1.4 μM; Table S1). In addition, the antioxidant assays of the isolated compounds were carried out by DPPH radicals scavenging and FRAP models. The results revealed that **15** exhibited significant DPPH radical scavenging activity with the IC_50_ value of 34.1 μM and displayed strong reduction of Fe^3+^ with the FRAP value of 9.0 mM under the concentration of 3.1 μg/mL; thus, **15** was more potent than the positive control ascorbic acid (DPPH, IC_50_ = 115.1 μM; FRAP = 5.6 mM under 3.1 μg/mL; Table S2). However, the radical scavenging effects of **1**–**14** were less than 50% under the concentration of 50 μg/mL.

## 3. Materials and Methods

### 3.1. General Experimental Procedures

The Optical rotations were measured on a JASCO P-1020 digital polarimeter (Jasco Corp., Tokyo, Japan). UV spectra were recorded by a Milton Roy UV–VIS spectrophotometer (Hitachi, Tokyo, Japan). IR spectra were performed on a Nicolet-Nexus-470 spectrometer using KBr pellets (Thermo Electron, Waltham, MA, USA). NMR spectra were tested by a JEOL JEMECP NMR spectrometer (600 MHz for ^1^H NMR, 150 MHz for ^13^C NMR and 500 MHz for NOE spectra, JEOL, Tokyo, Japan) using tetramethylsilane (TMS) as an internal standard. ESIMS spectra were measured on a Micromass Q-TOF spectrometer (Waters Corp., Manchester, UK). ECD spectra were obtained on a JASCO J-815 circular dichroism spectrometer (JASCO Electric Co., Ltd., Tokyo, Japan). In the biological assay, the optical densities (OD) were acquired by a multimode reader Spark 10M (Tecan, Männedorf, Switzerland). Semipreparative HPLC was performed on a Hitachi L-2000 HPLC system coupled with a Hitachi L-2455 photodiode array detector and a Kromasil C_18_ semipreparative HPLC column (250 mm × 10 mm, 5 μm). Silica gel (Qingdao Haiyang Chemical Group Co., Qingdao, China) and Sephadex LH-20 (Amersham Biosciences Inc., Piscataway, NJ, USA) were used for column chromatography (CC). Precoated silica gel GF254 plates (Yantai Zifu Chemical Group Co., Yantai, China) were used for thin layer chromatography (TLC).

### 3.2. Fungal Material

The fungal strain *A. versicolor* SH0105 was isolated from a deep-sea sediment sample collected at a depth of 5455 m from the Mariana Trench. The strain was deposited in the Key Laboratory of Marine Drugs, the Ministry of Education of China, School of Medicine and Pharmacy, Ocean University of China, Qingdao, China. The fungal strain was identified as *A. versicolor* according to its morphological features, amplification and sequencing of the DNA sequences of the ITS region, and construction of phylogenetic tree by MEGA 7.0 (Temple University, Philadelphia, PA, USA; Figure S1). The sequence data was submitted to NCBI with the GeneBank accession number MT620963.

### 3.3. Fermentation, Extraction, and Isolation

The fungal strain was cultured on rice solid medium (100 × 1000 mL Erlenmeyer flasks, each containing 80 g of rice and 80 mL of sea water) for 60 days at 25 °C. The fermented rice substrate was extracted three times with ethyl acetate (EtOAc) and concentrated under the vacuum evaporation to yield an organic extract (85 g). Then, the extract was performed on the silica gel vacuum liquid chromatography (VLC) eluting by a gradient of petroleum ether (PE)—EtOAc (100%, 90%, 70%, 50%, and 0% PE) and 10% EtOAc-MeOH to give six fractions (Fr.1–Fr.6). Fr.3 was subjected to the octadecyl silane (ODS) column with MeOH-H_2_O (15–100%) to afford four subfractions (Fr.3A–Fr.3D). Fr.3A was separated by Sephadex LH-20 column chromatography (CC) eluting with MeOH and then purified by semipreparative HPLC (75% MeOH-H_2_O) to obtain **1** (2 mg), **2** (4 mg), **3** (18 mg), and **4** (4 mg). Fr.3B was repeatedly isolated by silica gel CC eluting with PE-EtOAc to produce **12** (10 mg) and **13** (8 mg). Fr.3C was subjected to Sephadex LH-20 CC and then performed on HPLC (60% MeOH-H_2_O) to yield **14** (5 mg) and **15** (38 mg). Fr.3D was chromatographed on silica gel CC and recrystallized to give **8** (6 mg) and **9** (12 mg). Fr.4 was also fractionated on ODS column with MeOH-H_2_O (15–100%) to obtain three subfractions (Fr.4A–Fr.4C). Fr.4A was subjected to Sephadex LH-20 CC (50% CH_2_Cl_2_-MeOH) to obtain two subfractions (Fr.4A1–Fr.4A2). Fr.4A1 was purified on HPLC (45% MeOH-H_2_O) to yield **7** (14 mg). Fr.4A2 was reseparated by silica gel CC and HPLC (60% MeOH-H_2_O) to provide **5** (65 mg) and **6** (26 mg). Fr.4B was isolated on silica gel CC and further eluted with Sephadex LH-20 CC (50% CH_2_Cl_2_-MeOH) to afford **9** (6 mg) and **11** (9 mg).

Isoversiol F (**1**): yellowish oil; ${\left[\alpha \right]}_{D}^{20}$ − 11 (*c* 0.1, MeOH); UV (MeOH) *λ*_max_ (log *ε*) 236 (1.48) nm; IR (KBr) *v*_max_ 3734, 2360, 1699, 1539, 1033 cm^−1^; ^1^H and ^13^C NMR see Table 1; HRESIMS *m*/*z* 261.1492 [M + H]^+^ (calcd for C_16_H_21_O_3_, 261.1485).

Decumbenone D (**2**): yellowish oil; ${\left[\alpha \right]}_{D}^{20}$+70.8 (*c* 0.1, MeOH); UV (MeOH) *λ*_max_ (log *ε*) 283 (1.93) nm; IR (KBr) *v*_max_ 3444, 2958, 2360, 1687, 1380, 1113 cm^−1^; ^1^H and ^13^C NMR see Table 1; HRESIMS *m*/*z* 233.1543 [M + H_2_O + H]^+^ (calcd for C_15_H_21_O_2_, 233.1536).

Palitantin B (**7**): yellow solid; ${\left[\alpha \right]}_{D}^{20}$+53.2 (*c* 0.1, MeOH); UV (MeOH) *λ*_max_ (log *ε*) 321(2.66) nm; IR (KBr) *v*_max_ 3748, 2361, 1658, 1598, 987 cm^−1^; ^1^H and ^13^C NMR see Table 1; HRESIMS *m*/*z* 253.1442 [M + H]^+^ (calcd for C_14_H_21_O_4_, 253.1434); 275.1262 [M + Na]^+^ (calcd for C_14_H_20_O_4_Na, 275.1254).

1,3-Di-*O*-methyl-norsolorinic acid (**8**): red powder; UV (CHCl_3_) *λ*_max_ (log *ε*) 245 (1.24), 260 (1.05), 340 (0.65) nm; IR (KBr) *v*_max_ 3362, 2362, 1683, 1423, 1059 cm^−1^; ^1^H and ^13^C NMR see Table 2; HRESIMS *m*/*z* 397.1282 [M + H]^+^ (calcd for C_22_H_21_O_7_, 397.1293).

### 3.4. ECD Calculations

The Merck molecular force field (MMFF94S) was used to conformational searches of compounds **1**–**2** and **6**–**7** during theoretical ECD calculations. All conformers were optimized twice by the basis set at the B3LYP/6-31G (d) and B3LYP/6-311+G (d) levels using the Gaussian 09 (Gaussian Inc., Wallingford, CT, USA) [39]. The ECD spectrum was calculated by the time-dependent density functional theory (TD-DFT) method at B3LYP/6-311++G (2d, p) level and simulated by Boltzmann distributions in SpecDis 1.62 (University of Würzburg, Würzburg, Germany) [40].

### 3.5. Biological Assays

#### 3.5.1. Antimicrobial Assay

The antimicrobial assays were evaluated using a broth microdilution method in 96-well polystyrene microtiter plates Costar 3599 (Corning Inc., New York, NY, USA) according to the standard of Clinical and Laboratory Standards Institute (CLSI) [41]. Three pathogenic bacterial strains, *Staphylococcus aureus* (ATCC 27154), *Escherichia coli* (ATCC 25922), and *Pseudomonas aeruginosa* (ATCC 10145); five fouling bacterial strains, *P. fulva* (ATCC 31418), *Aeromonas salmonicida* (ATCC 7965D), *Photobacterium angustum* (ATCC 33975), *Enterobacter cloacae* (ATCC 39978), and *E. hormaechei* (ATCC 700323); and one pathogenic fungal strain *Candida albicans* (ATCC 76485) were used as the test strains. First, the tested pathogenic bacteria, fouling bacteria, and pathogenic fungus were inoculated in 10 mL of LB (yeast extract 5 g/L, peptone 10 g/L, NaCl 10 g/L), 2216E (Hopebio, Qingdao, China), and YM (Hopebio, Qingdao, China) liquid medium, respectively, and cultivated at 37 °C for 12 h to yield the initial microbial liquids. The microbial density was adjusted to 0.5 MacFarland and then diluted 1000 times using the corresponding broth to obtain the tested microbial suspension with an inoculum density of 1 ×10^5^ cfu/mL. The tested compounds were dissolved in 100% DMSO to obtain the mother solution with the initial concentration of 1 mg/mL. Following the principle of twofold serial dilution, each well contained 5 µL of tested compounds and 195 µL of the microbial suspension to obtain the final measured concentration of 25–0.098 μg/mL. Finally, the plates were incubated at 37 °C for 24 h and the optical density of each well was recorded by microplate reader (Tecan, Männedorf, Switzerland) at 600 nm. MIC represents the minimal inhibitory concentration of compound without visible microbial growth. The antimicrobial assays were performed in triplicate. Broad-spectrum antimicrobial ciprofloxacin and commercial antifouling sea-nine 211 were used as positive controls for pathogenic and fouling microbial strains, respectively. DMSO was used as a negative control.

#### 3.5.2. Antioxidant Activity

The DPPH radical scavenging assay and ferric reducing antioxidant power assay (FRAP) were used to evaluate the antioxidant activities of the isolated compounds [42]. The samples and positive control ascorbic acid were dissolved in DMSO with final concentrations of 100, 50, 25, 12.5, and 6.25 μg/mL. DPPH was dissolved in anhydrous ethanol (EtOH) with the concentrations of 0.05 mg/mL. Fe^3+^-TPTZ solution consisted of 2 mmol/L FeCl_3_ and 2,4,6-Tris(2-pyridyl)-s-triazine (TPTZ), respectively. Tested samples (100 μL) were added to 100 μL of fresh DPPH or Fe^3+^-TPTZ solution, then reacted in the dark for 30 min. The optical density (OD) was measured by a multimode reader Spark 10 M (Tecan, Männedorf, Switzerland) at 517 and 593 nm, respectively. The EtOH and DMSO were employed as a blank and negative control, respectively. The IC_50_ values were calculated on the software of GraphPad Prism 5 (GraphPad Software Inc., San Diego, CA, USA).

## 4. Conclusions

Deep-sea derived fungi are potential resources to seek for structural novel and diverse biological natural products. In the present study, chemical investigation of the deep-sea-derived fungus *A. versicolor* SH0105 led to the isolation of four new polyketides (**1**–**2** and **7**–**8**), along with 11 known compounds (**3**–**6** and **9**–**15**), which enriched the diversity of secondary metabolites from the deep-sea-derived *Aspergillus*. The structures and absolute configurations of new compounds were elucidated by comprehensive spectroscopic data and ECD calculations, and it is the first time to determine the absolute configuration of known decumbenone A (**6**). In the bioactive assays, compound **14** displayed obvious inhibitory activity against *S. aureus* (ATCC 27154) and **15** exhibited significant DPPH radical scavenging activity and displayed strong reduction of Fe^3+^, which were more potent than ascorbic acid, indicating the prospect to discovery of chemical entities with antimicrobial and antioxidant activities from the deep-sea medicinal microbial resources.

## Figures and Tables

**Table 1:** The ^1^H and ^13^C NMR data of **1**–**2** and **7.**

No.	1 ^a^		2 ^b^		7 ^b^	
	*δ*_C_, Type	*δ*_H_ (*J* in Hz)	*δ*_C_, type	*δ*_H_ (*J* in Hz)	*δ*_C_, Type	*δ*_H_ (*J* in Hz)
1	67.5, CH	5.20, m	67.3, CH	4.22, m	199.6, C	
2	38.8, CH_2_	2.00, m; 1.30, m	40.8, CH_2_	1.85, m; 1.22, m	76.8, CH	4.31, d (3.0)
3	25.9, CH	2.56, m	26.9, CH	2.53, m	70.9, CH	4.41, m
4	136.6, CH	5.71, brs	134.9, CH	5.58, brs	33.8, CH_2_	3.01, dd (18.2, 3.5)2.90, dd (18.2, 3.5)
5	130.3, C		132.7, C		151.0, C	
6	130.5, CH	6.00, d (9.8)	129.6, CH	5.94, d (9.8)	132.5, C	
7	127.6, CH	5.64, d (9.8)	134.1, CH	5.35, d (9.8)	54.5, CH_2_	4.40, d (11.6)4.58, d (11.6)
8	86.2, C		75.2, C		128.4, CH	6.88, m
9	49.4, C		58.9, C		139.3, CH	6.87, m
10	41.9, CH	2.65, q (3.3)	43.3, CH	2.88, q (3.3)	132.3, CH	6.33, ddq (15.1, 8.4, 1.4)
11	200.2, C		216.3, C		142.3, CH	6.11, dt (15.1, 7.2)
12	106.5, CH	5.31, d (5.9)	30.8, CH_3_	2.28, s	36.1, CH_2_	2.19, dq (7.2, 1.4)
13	158.4, CH	7.05, d (5.9)	26.5, CH_3_	1.12, s	23.3, CH_2_	1.50, qt (7.4, 1.4)
14	21.0, CH_3_	1.47, s	15.2, CH_3_	1.44, s	14.0, CH_3_	0.96, t (7.4)
15	17.2, CH_3_	1.28, s	21.6, CH_3_	1.02, d (7.1)		
16	21.2, CH_3_	1.04, d (7.1)				

^a, b^ Recorded at 600 MHz for ^1^H NMR and 150 MHz for ^13^C NMR in CDCl_3_ and MeOH-*d*_4_, respectively.

**Table 2:** The ^1^H and ^13^C NMR data of **8** in DMSO-*d*_6._

No.	*δ*_C_, type	*δ*_H_ (*J* in Hz)	No.	*δ*_C_, type	*δ*_H_ (*J* in Hz)
1	163.1, C		13	183.5, C	
2	104.6, CH	6.99, d (2.5)	14	113.8, C	
3	164.9, C		15	203.2, C	
4	104.9, CH	7.24, d (2.5)	16	43.7, CH_2_	2.74, t (7.3)
5	133.7, C		17	22.6, CH_2_	1.54, dd (8.5, 5.6)
6	181.8, C		18	30.7, CH_2_	1.25, m
7	136.4, C		19	21.9, CH_2_	1.25, m
8	106.6, CH	7.08, s	20	13.8, CH_3_	0.83, t (7.0)
9	163.0, C		1-OCH_3_	56.6, CH_3_	3.91, s
10	121.6, C		3-OCH_3_	56.2, CH_3_	3.99, s
11	161.3, C		11-OH		13.9, s
12	109.1, C				

## Supplementary Materials

The following are available online at https://www.mdpi.com/1660-3397/18/12/636/s1. Figure S1: The neighbor-joining phylogenetic tree of the fungus *A. versicolor* SH0105: Figure S2 to Figure S31: The HRESIMS and 1D and 2D NMR spectra of new compounds **1**–**2** and **7**–**8**; Figure S32 to Figure S35: The lowest-energy conformers of compounds **1**–**2** and **6**–**7** in ECD calculations; Table S1 and Table S2: The data of antimicrobial and antioxidant activities.
