# Supplementary material for: Antimicrobial and Antioxidant Polyketides from a Deep-Sea-Derived Fungus Aspergillus versicolor SH0105
Source: Mar Drugs. 2020 Dec 11;18(12):636. doi: 10.3390/md18120636 (PMC7764742; doi:10.3390/md18120636)
Supplement: Supplementary file 1 [file marinedrugs-18-00636-s001.pdf]

Supplementary Materials
Antimicrobial and antioxidant polyketides from a deep-sea-derived fungus *Aspergillus versicolor* SH0105

Lu-Jia Yang[1,2], Xiao-Yue Peng[1,2], Ya-Hui Zhang[1,2], Zhi-Qing Liu[1,2], Xin Li[1,2], Yu-Cheng Gu[3], Chang-Lun Shao[1,2], Zhuang Han[4,*], and Chang-Yun Wang[1,2,*]

1 Key Laboratory of Marine Drugs, The Ministry of Education of China, School of Medicine and Pharmacy; Institute of Evolution & Marine Biodiversity, Ocean University of China, Qingdao 266003, China; yanglujia@stu.ouc.edu.cn (L.-J. Y.); pengxiaoyue@stu.ouc.edu.cn (X.-Y.P.); zhangyahui@stu.ouc.edu.cn (Y.-H.Z.); liuzhiqing@ouc.edu.cn (Z.-Q.L.); lixin8962@ouc.edu.cn (X.L.); shaochanglun@ouc.edu.cn (C.-L.S.)

2 Laboratory for Marine Drugs and Bioproducts, Qingdao National Laboratory for Marine Science and Technology, Qingdao 266237, China;

3 Jealott's Hill International Research Centre, Syngenta, Bracknell, Berkshire RG42 6EY, UK; yucheng.gu@syngenta.com

4 Institute of Deep-sea Science and Engineering, Chinese Academy of Science, Sanya 572000, China

* Correspondence: changyun@ouc.edu.cn (C.-Y.W.); zhuanghan@idsse.ac.cn (Z.H.); Tel.: +86-0532-8203-1536 (C.-Y.W.); Tel.: +86-0898-88215868 (Z.H.)

List of Supporting Information

DNA sequences of the ITS region of the fungus *Aspergillus versicolor* SH0105

Figure S1 The neighbor-joining phylogenetic tree of the fungus *Aspergillus versicolor* SH0105

Figure S2 The ^{1}H NMR (600 MHz, $CDCl_3$) spectrum of isoversiol F (**1**)

Figure S3 The ^{13}C NMR (150MHz, $CDCl_3$) spectrum of isoversiol F (**1**)

Figure S4 The HSQC ($CDCl_3$) spectrum of isoversiol F (**1**)

Figure S5 The ^{1}H-^{1}H COSY ($CDCl_3$) spectrum of isoversiol F (**1**)

Figure S6 The HMBC ($CDCl_3$) spectrum of isoversiol F (**1**)

Figure S7 The NOSEY ($CDCl_3$) spectrum of isoversiol F (**1**)

Figure S8 The NOE (500 MHz, $CDCl_3$) spectrum of isoversiol F (**1**)

Figure S9 The HRESIMS spectrum of isoversiol F (**1**)

Figure S10 The ^{1}H NMR (600 MHz, MeOH-d_4) spectrum of decumbenone D (**2**)

Figure S11 The ^{13}C NMR (150 MHz, MeOH-d_4) spectrum of decumbenone D (**2**)

Figure S12 The HSQC (MeOH-d_4) spectrum of decumbenone D (**2**)

Figure S13 The ^{1}H-^{1}H COSY (MeOH-d_4) spectrum of decumbenone D (**2**)

Figure S14 The HMBC (MeOH-d_4) spectrum of decumbenone D (**2**)

Figure S15 The NOSEY (MeOH-d_4) spectrum of decumbenone D (**2**)

Figure S16 The ^{1}H NMR (500 MHz, $CDCl_3$) spectrum of decumbenone D (**2**)

Figure S17 The NOE (500 MHz, $CDCl_3$) spectrum of decumbenone D (**2**)

Figure S18 The HRESIMS spectrum of decumbenone D (**2**)

Figure S19 The ^{1}H NMR (600 MHz, MeOH-d_4) spectrum of palitantin B (**7**)

Figure S20 The ^{13}C NMR (150 MHz, MeOH-d_4) spectrum of palitantin B (**7**)

Figure S21 The HSQC (MeOH-d_4) spectrum of palitantin B (**7**)

Figure S22 The ^{1}H-^{1}H COSY (MeOH-d_4) spectrum of palitantin B (**7**)

Figure S23 The HMBC (MeOH-d_4) spectrum of palitantin B (**7**)

Figure S24 The NOSEY (MeOH-d_4) spectrum of palitantin B (**7**)

Figure S25 The HRESIMS spectrum of palitantin B (**7**)

Figure S26 The ^{1}H NMR (600 MHz, DMSO-d_6) spectrum of 1,3-di-*O*-methyl-norsolorinic acid (**8**)

Figure S27 The ^{13}C NMR (150 MHz, DMSO-d_6) spectrum of 1,3-di-*O*-methyl-norsolorinic acid (**8**)

Figure S28 The HSQC (DMSO-d_6) spectrum of 1,3-di-*O*-methyl-norsolorinic acid (**8**)

Figure S29 The ^{1}H-^{1}H COSY (DMSO-d_6) spectrum of 1,3-di-*O*-methyl-norsolorinic acid (**8**)

Figure S30 The HMBC (DMSO-d_6) spectrum of 1,3-di-*O*-methyl-norsolorinic acid (**8**)

Figure S31 The HRESIMS spectrum of 1,3-di-*O*-methyl-norsolorinic acid (**8**)

Figure S32 The lowest-energy conformer (1*S*,3*S*,8*R*,9*S*,10*S*)-**1** in ECD calculation

Figure S33 The lowest-energy conformer (1*S*,3*S*,8*R*,9*S*,10*S*)-**2** in ECD calculation

Figure S34 The lowest-energy conformer (2*R*,3*R*)-**7** in ECD calculation
Figure S35 The lowest-energy conformer (1*S*,3*S*,8*R*,9*R*,10*S*)-**6** in ECD calculation
Table S1 The antimicrobial activities of isolated compounds **14**–**15**
Table S2 The antioxidant activities of compound **15**

DNA sequences of the ITS region of the fungus *Aspergillus versicolor* SH0105

CTTCCGTAGGGTGAACCTGCGGAAGGATCATTACCGAGTGCGGGCTGCCTCCGGGCGCCCAA

CCTCCCACCCGTGACTACCTAACACTGTTGCTTCGGCGGGGAGCCCTTTCGGGGGCGAGCCG

CCGGGGACTACTGAACTTCATGCCTGAGAGTGATGCAGTCTGAGTCTGAATATAAAATCAGTC

AAAACTTTCAACAATGGATCTCTTGGTTCCGGCATCGATGAAGAACGCAGCGAACTGCGATA

AGTAATGTGAATTGCAGAATTCAGTGAATCATCGAGTCTTTGAACGCACATTGCGCCCCCTGG

CATTCCGGGGGGCATGCCTGTCCGAGCGTCATTGCTGCCCATCAAGCCCGGCTTGTGTGTTGG

GTCGTCGTCCCCCCCGGGGGACGGGCCCGAAAGGCAGCGGCGGCACCGTGTCCGGTCCTCG

AGCGTATGGGGCTTTGTCACCCGCTCGATTAGGGCCGGCCGGGCGCCAGCCGACGTCTCCAA

CCATTTTTTTCAGGTTGACCTCGGATCAGGTAGGGATACCCGCTGAACTTAAGCATATCAATA

AGCGGAGGAA

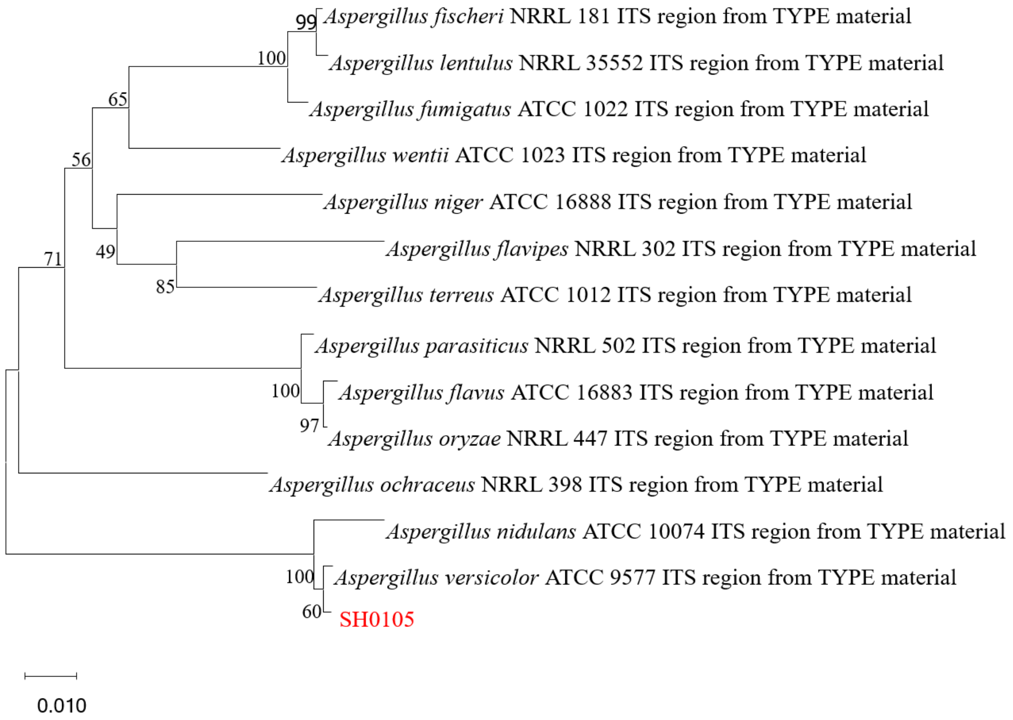

Figure S1 The neighbor-joining phylogenetic tree of the fungus *Aspergillus versicolor* SH0105

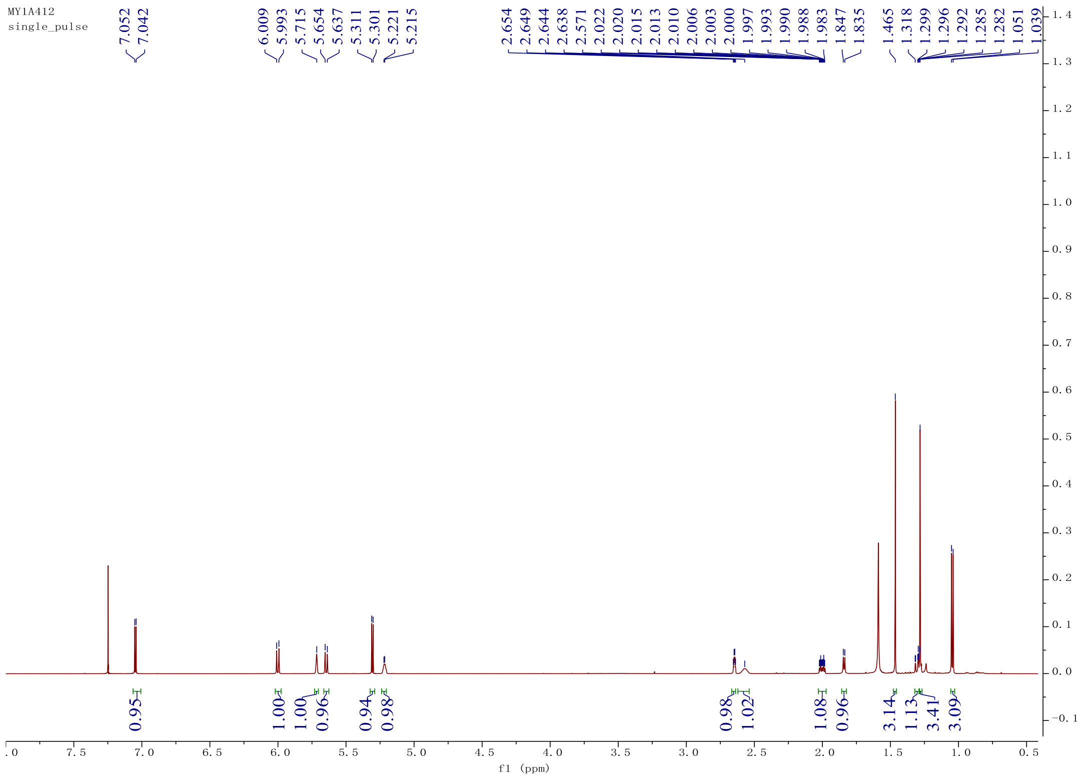

Figure S2 The ^{1}H NMR (600 MHz, $CDCl_3$) spectrum of isoversiol F (**1**)

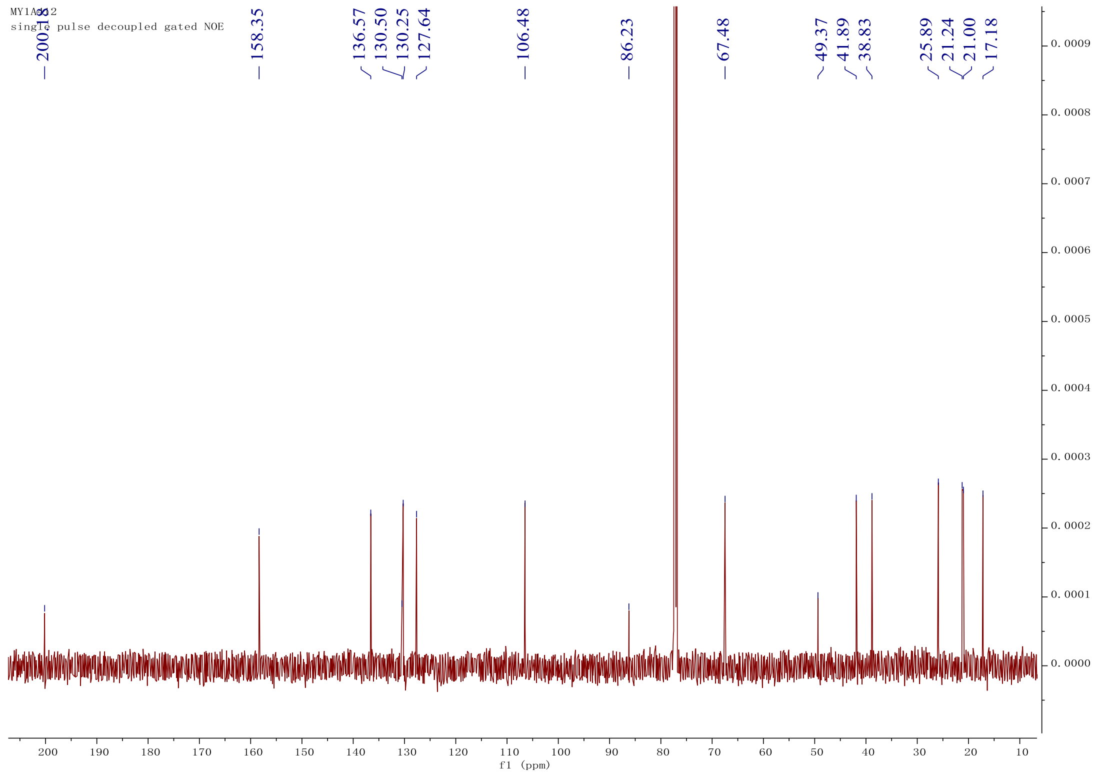

Figure S3 The ^{13}C NMR (150 MHz, $CDCl_3$) spectrum of isoversiol F (**1**)

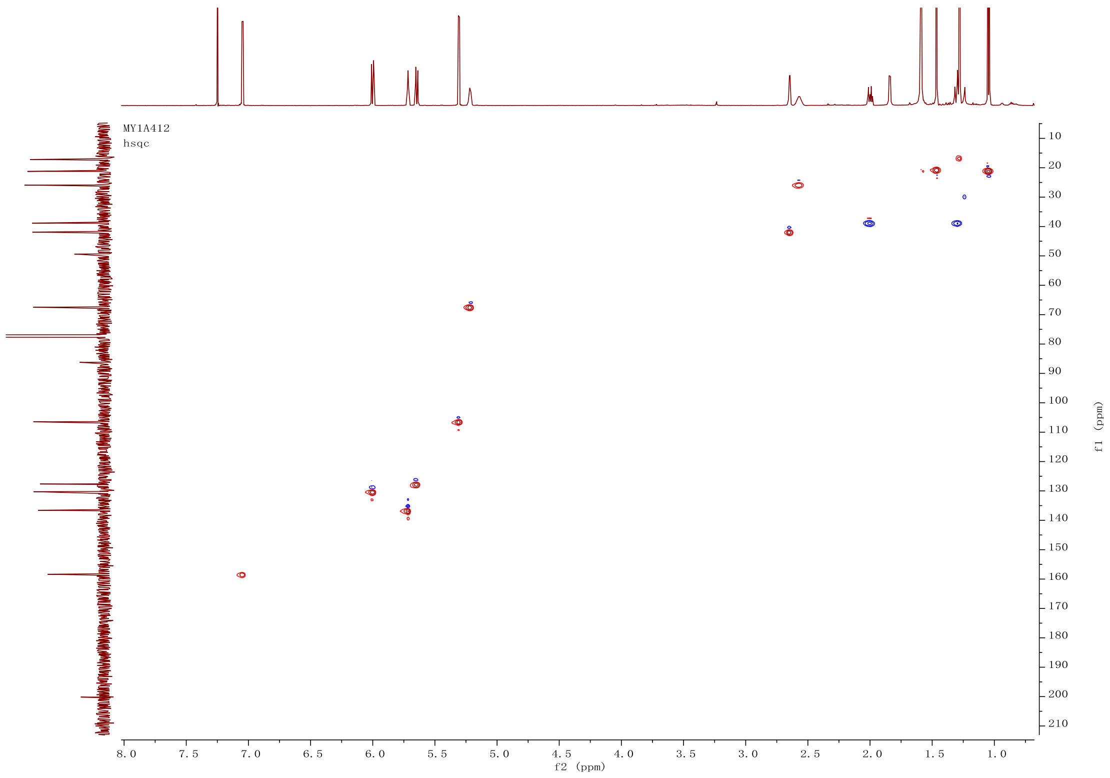

Figure S4 The HSQC ($CDCl_3$) spectrum of isoversiol F (**1**)

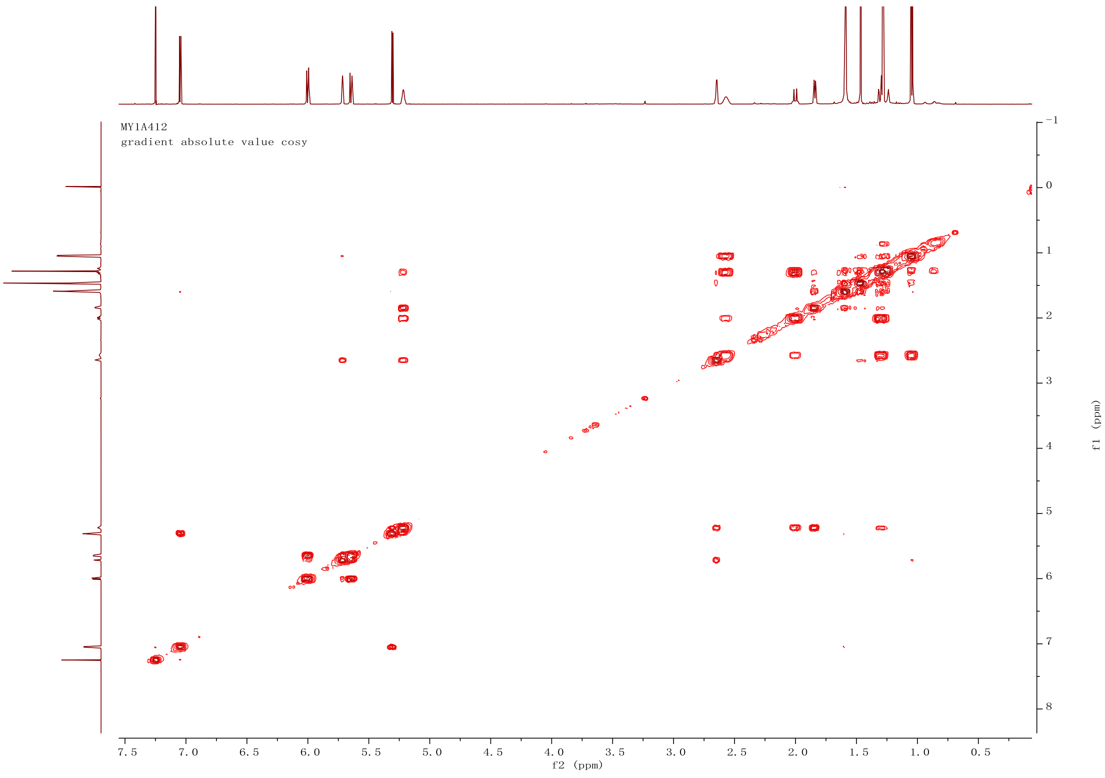

Figure S5 The ^1H-^1H COSY ($CDCl_3$) spectrum of isoversiol F (**1**)

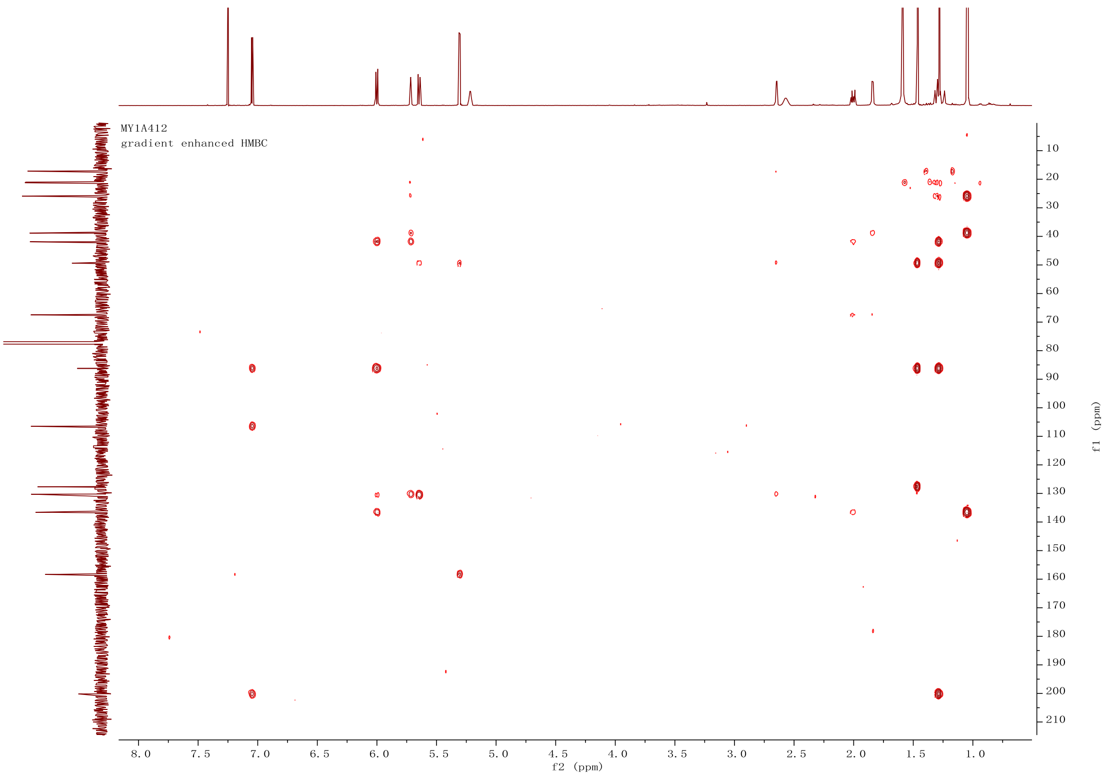

Figure S6 The HMBC ($CDCl_3$) spectrum of isoversiol F (**1**)

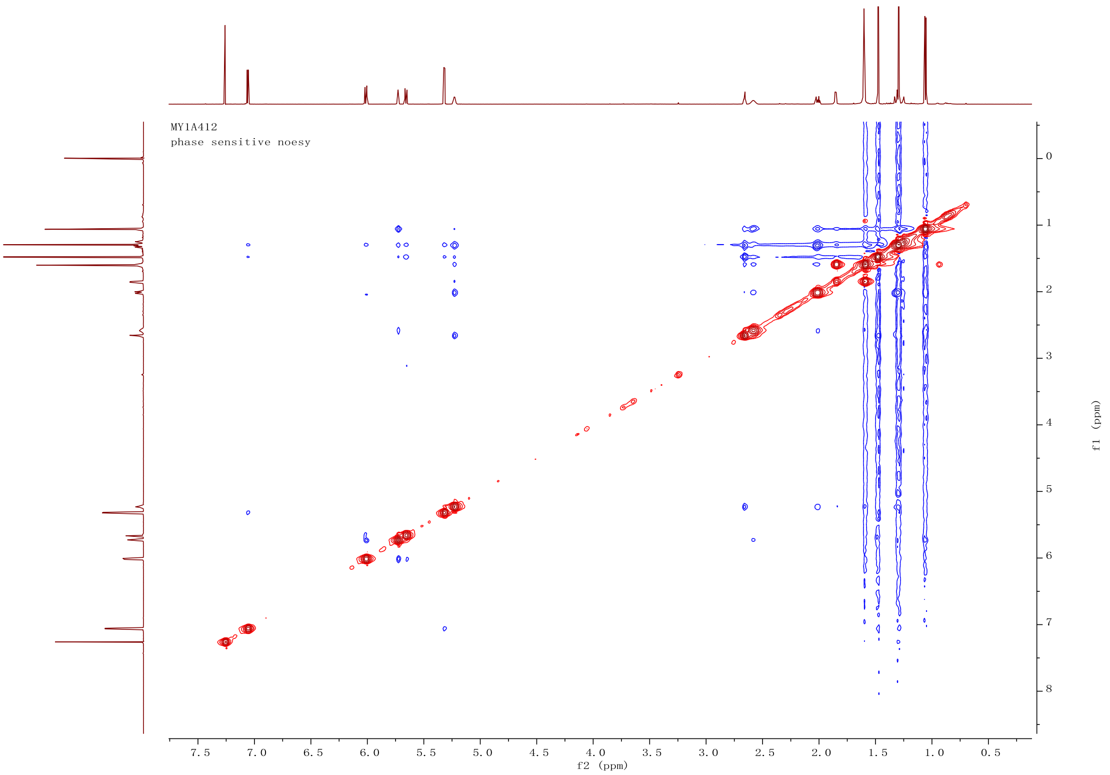

Figure S7 The NOSEY ($CDCl_3$) spectrum of isoversiol F (**1**)

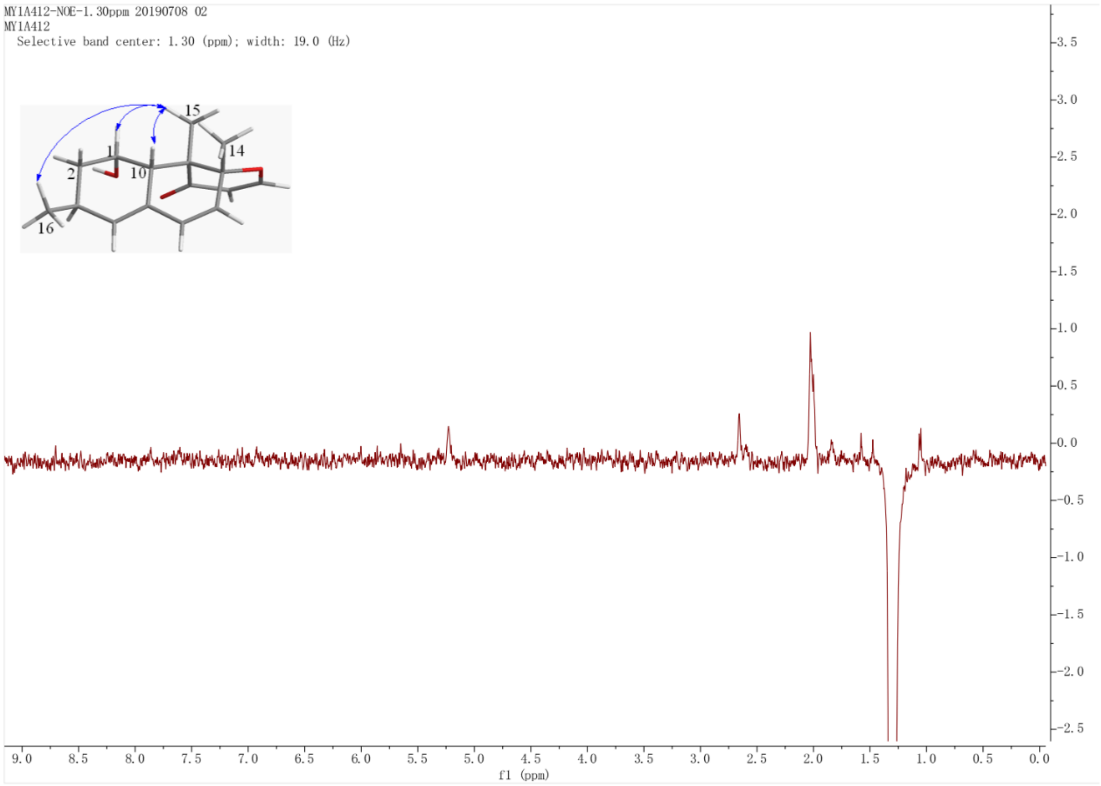

Figure S8 The NOE (500 MHz, $CDCl_3$) spectrum of isoversiol F (**1**)

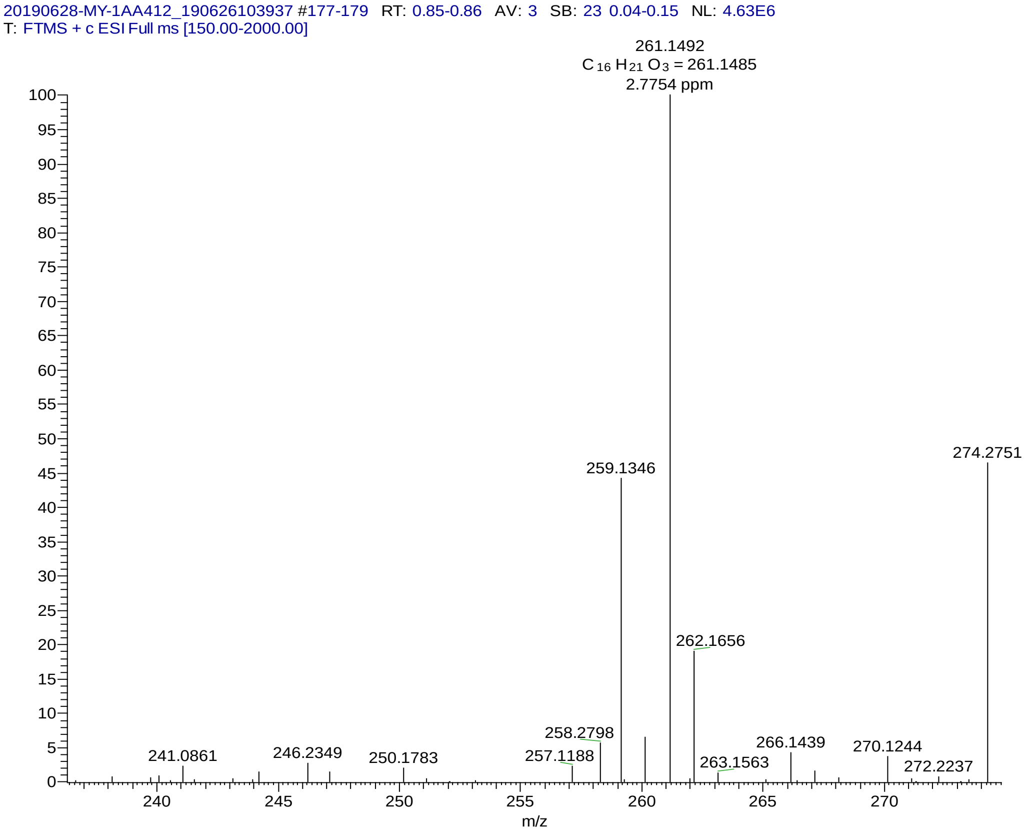

Figure S9 The HRESIMS spectrum of isoversiol F (**1**)

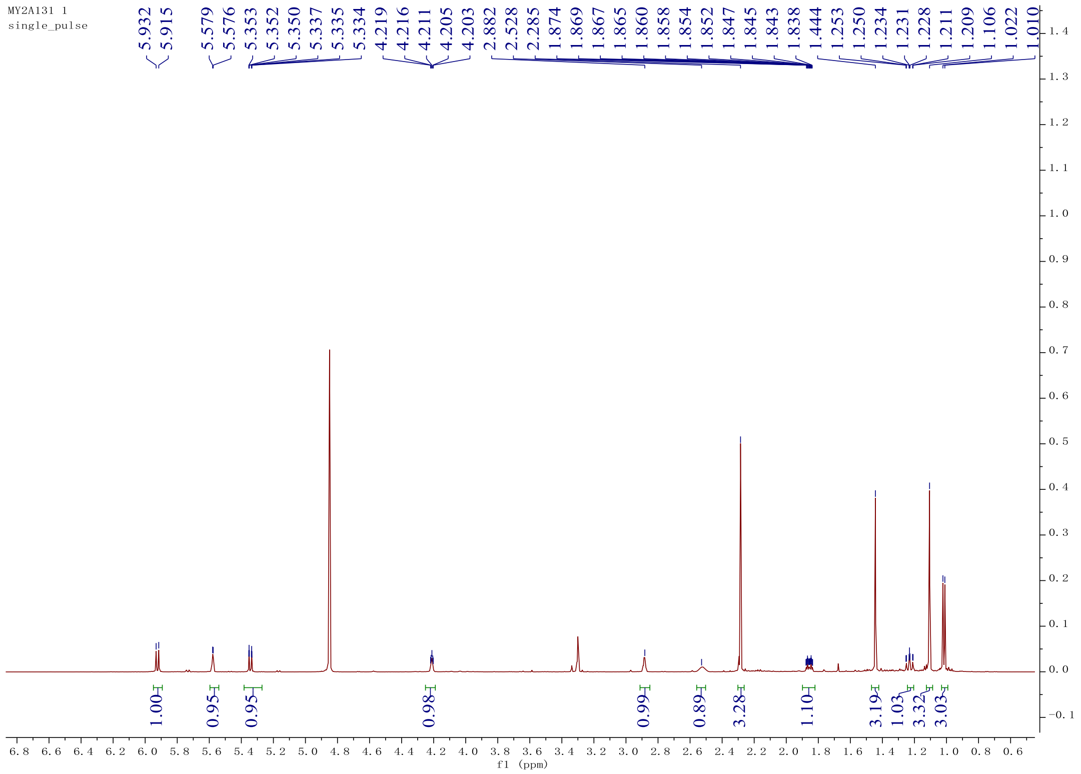

Figure S10 The ^{1}H NMR (600 MHz, MeOH-*d*4) spectrum of decumbenone D (**2**)

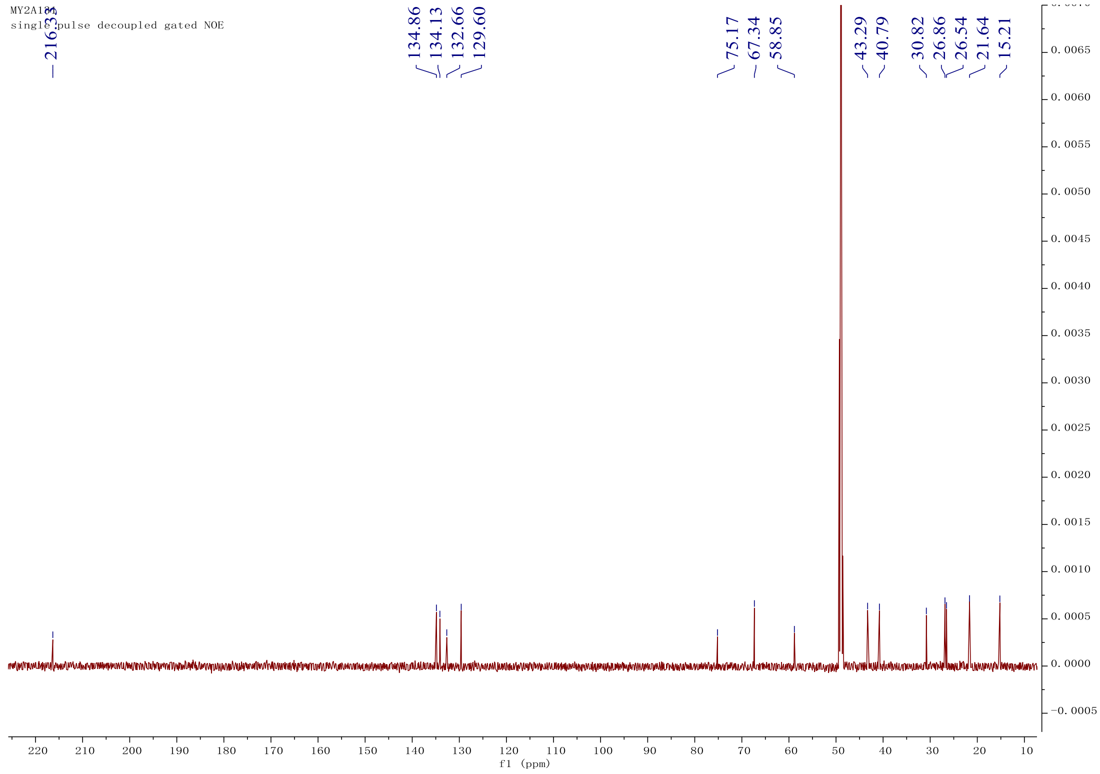

Figure S11 The^{13}C NMR (150MHz, MeOH-*d*4) spectrum of decumbenone D (**2**)

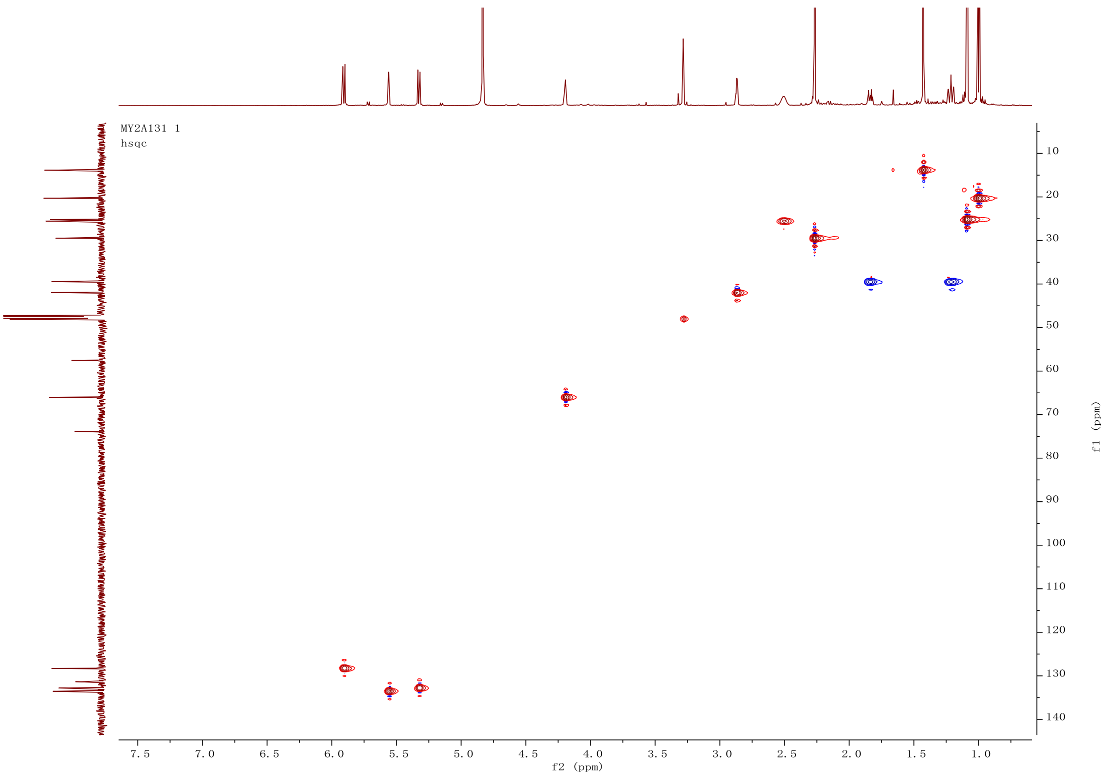

Figure S12 The HSQC (MeOH-*d*4) spectrum of decumbenone D (**2**)

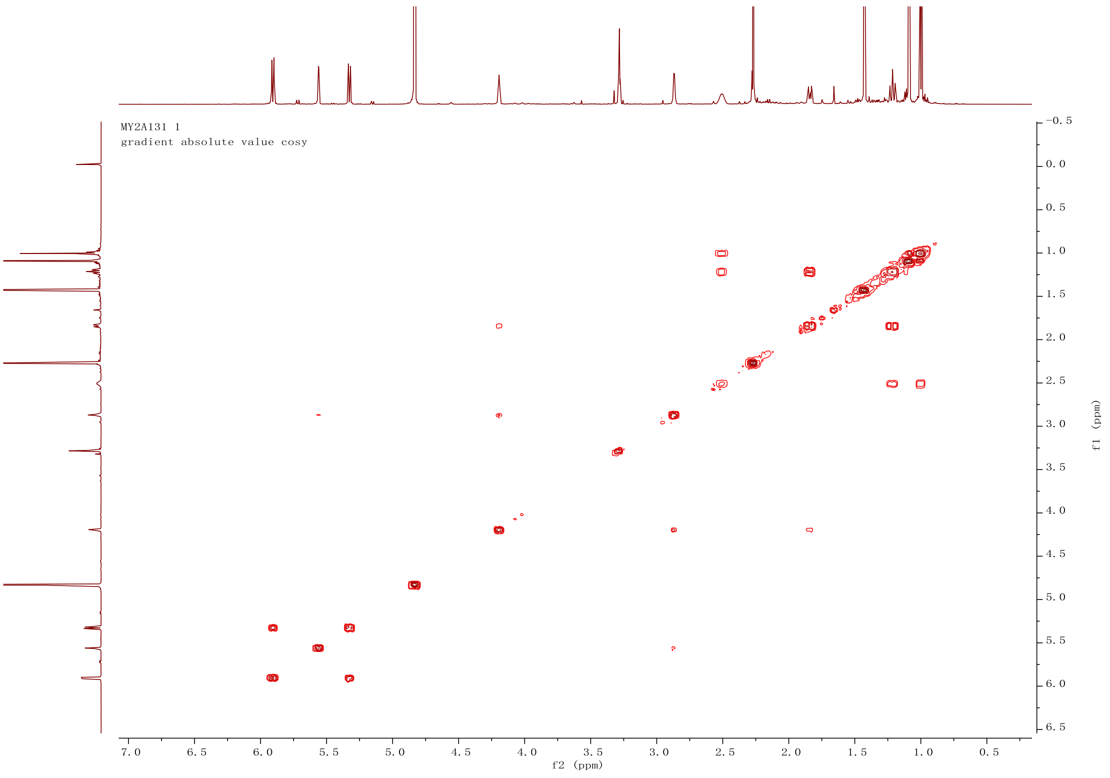

Figure S13 The ^{1}H-^{1}H COSY (MeOH-*d*4) spectrum of decumbenone D (**2**)

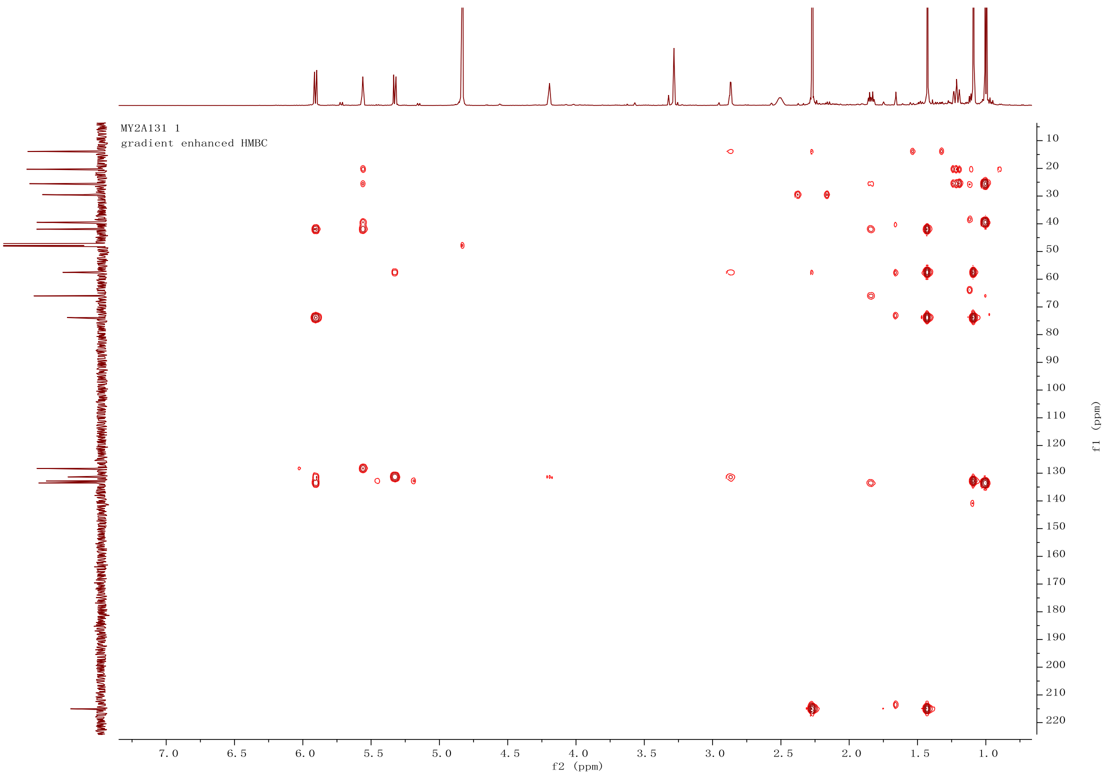

Figure S14 The HMBC (MeOH-d_4) spectrum of decumbenone D (**2**)

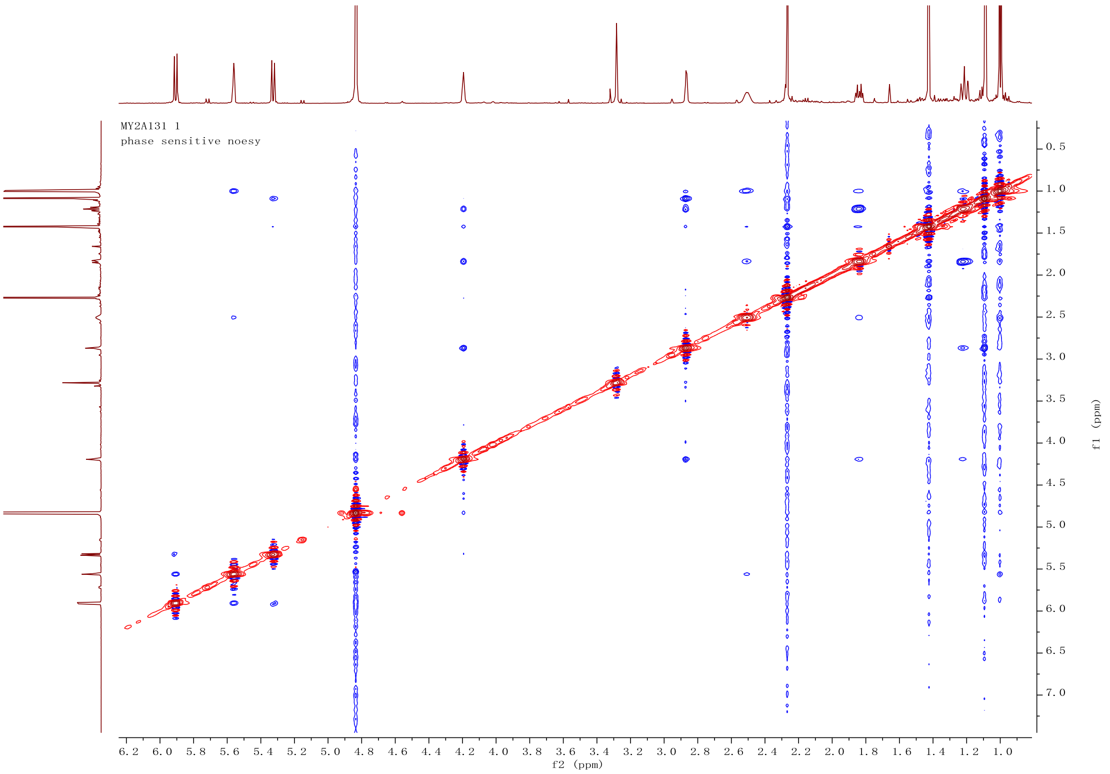

Figure S15 The NOSEY (MeOH-d_4) spectrum of decumbenone D (**2**)

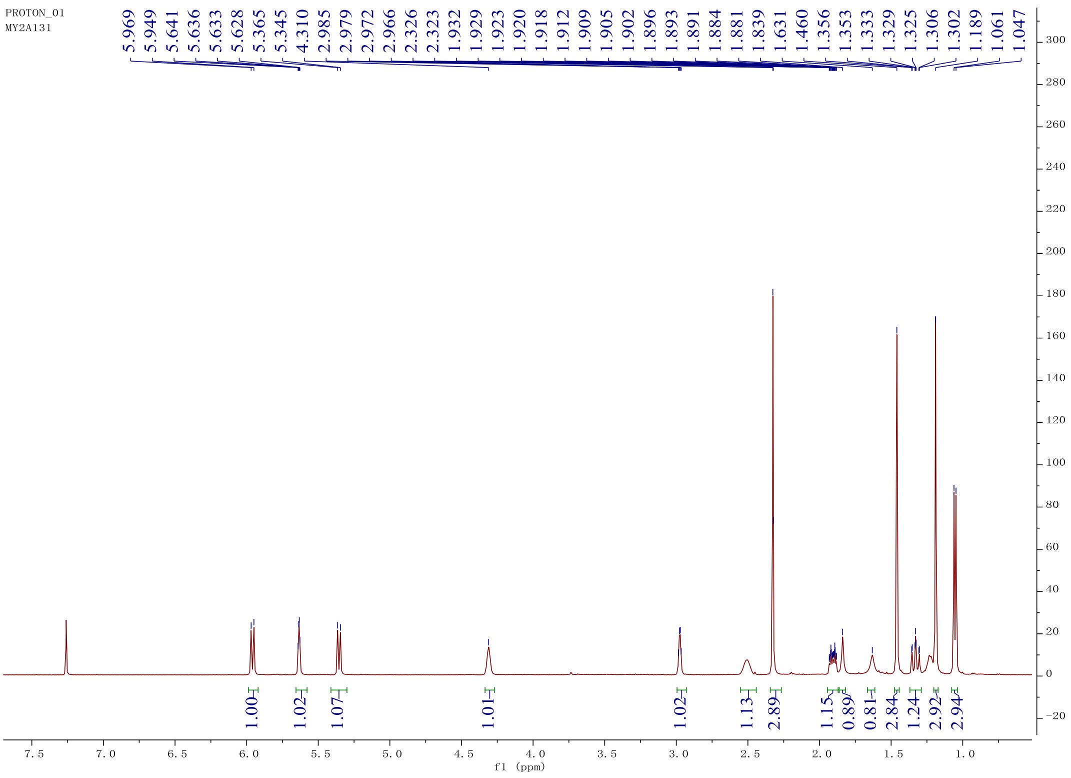

Figure S16 The ^{1}H NMR (500 MHz, $CDCl_3$) spectrum of decumbenone D (**2**)

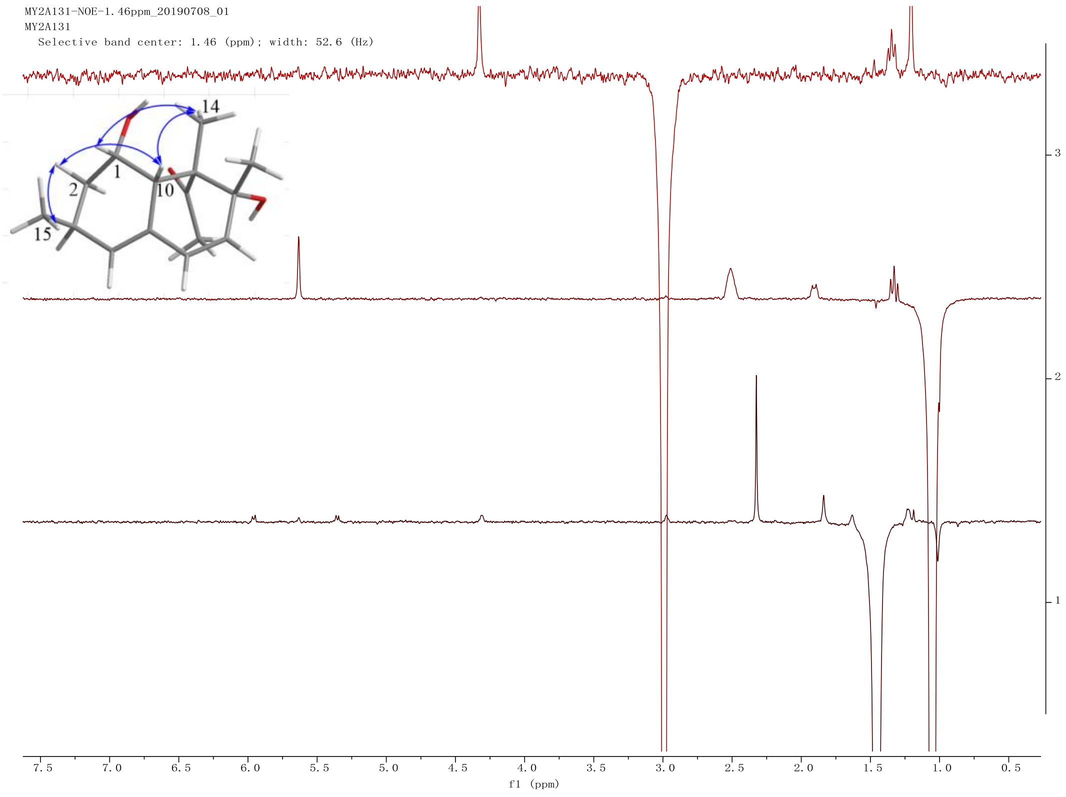

Figure S17 The NOE (500 MHz, $CDCl_3$) spectrum of decumbenone D (**2**)

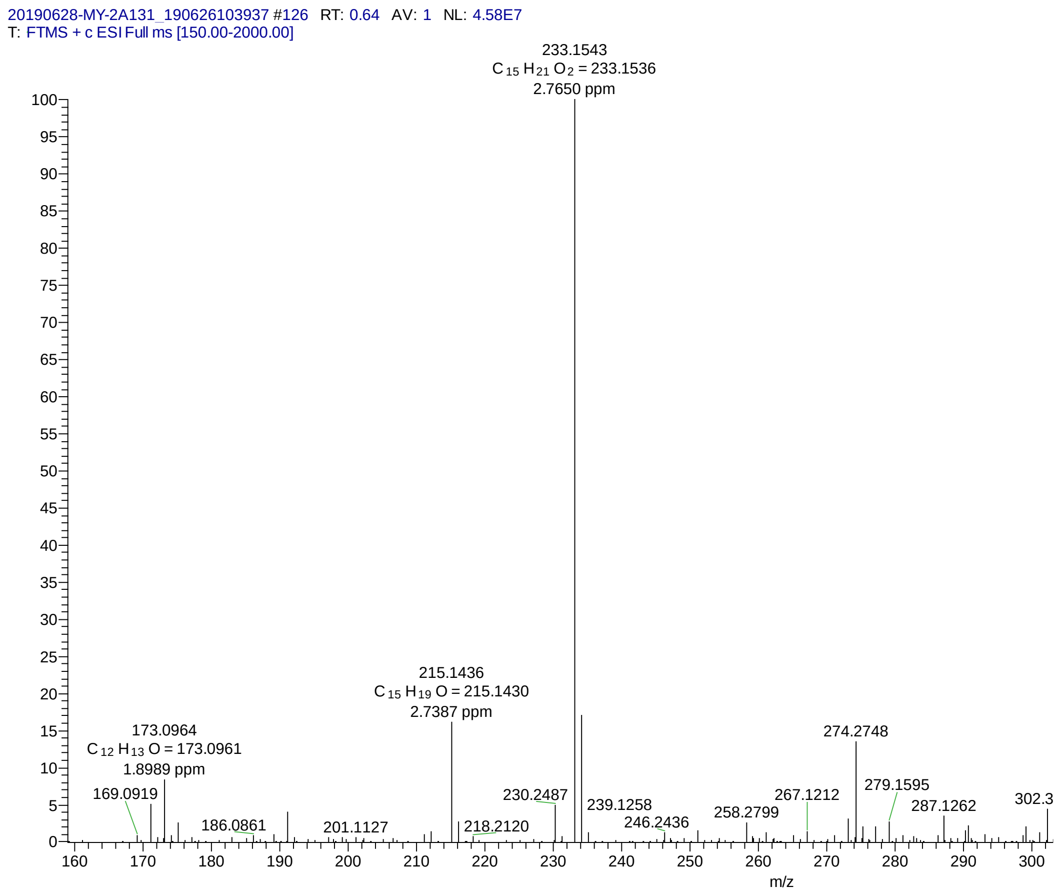

Figure S18 The HRESIMS spectrum of decumbenone D (**2**)

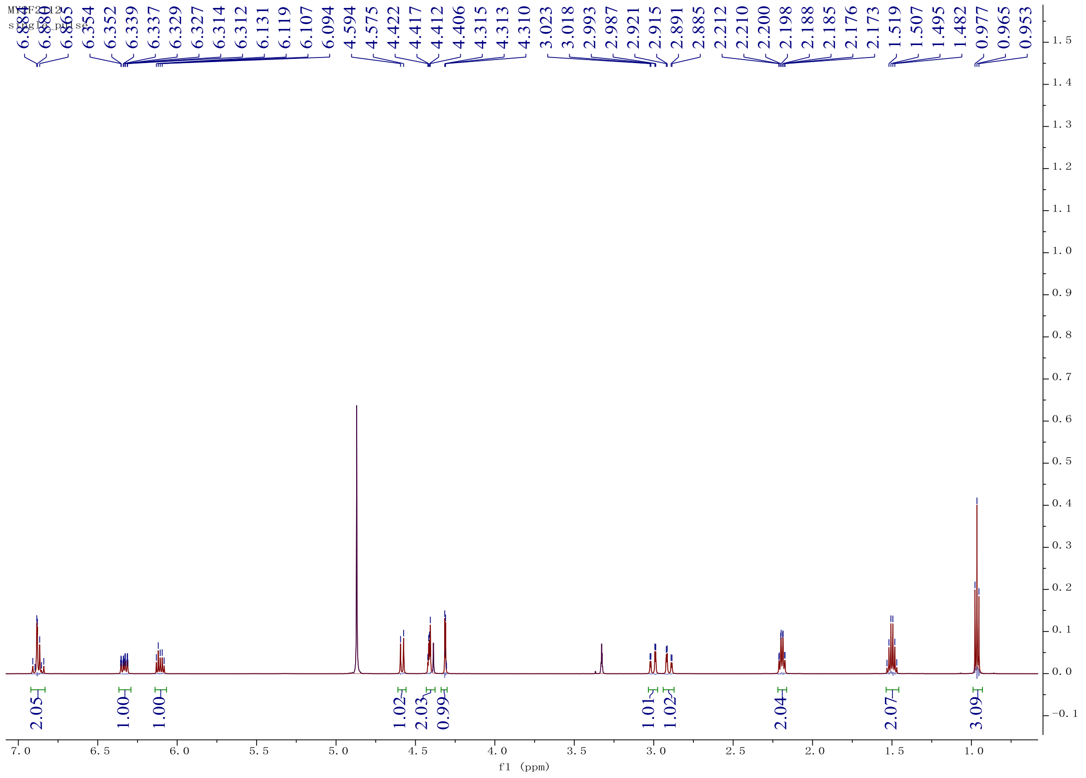

Figure S19 The ^{1}H NMR (600 MHz, MeOH-*d*$_4$) spectrum of palitantin B (**7**)

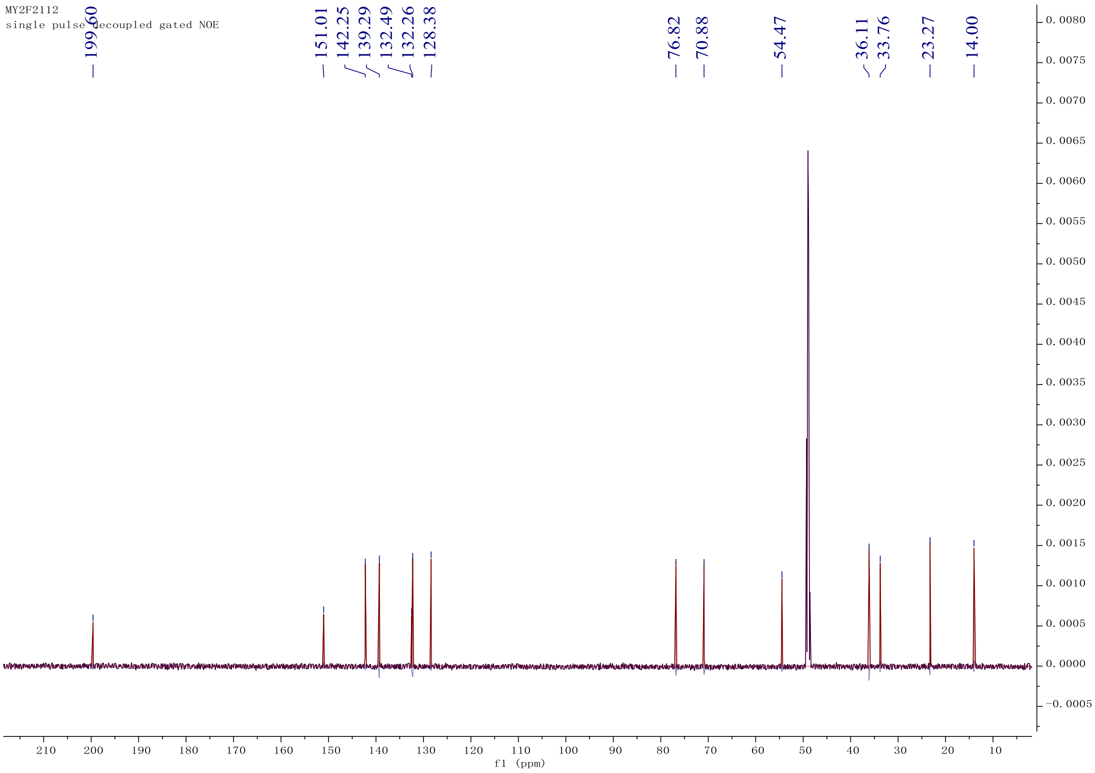

Figure S20 The ^{13}C NMR (150 MHz, MeOH-d_4) spectrum of palitantin B (**7**)

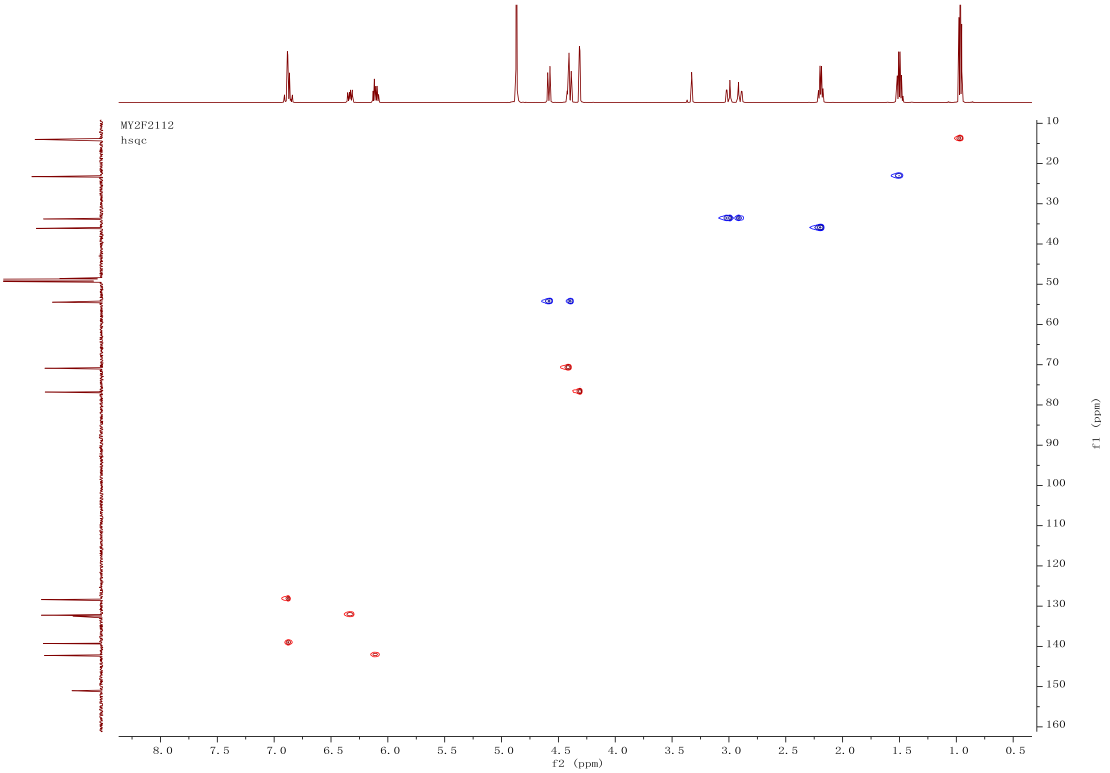

Figure S21 The HSQC (MeOH-d_4) spectrum of palitantin B (**7**)

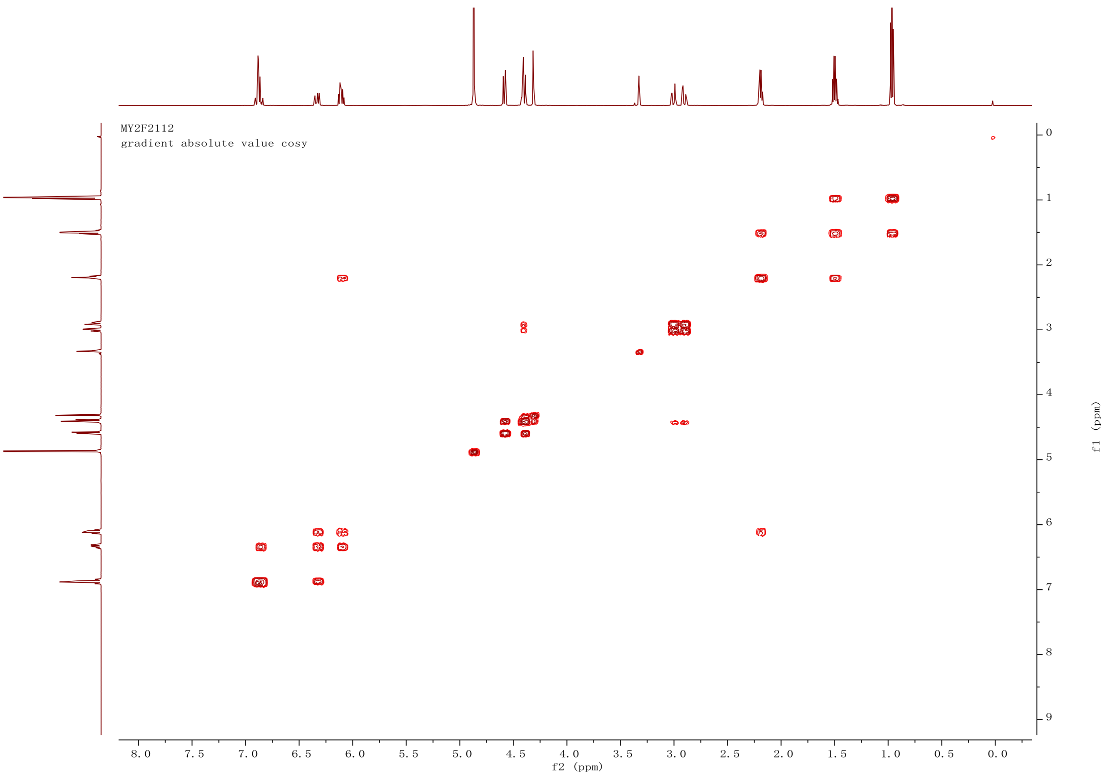

Figure S22 The ^1H-^1H COSY (MeOH-*d*$_4$) spectrum of palitantin B (**7**)

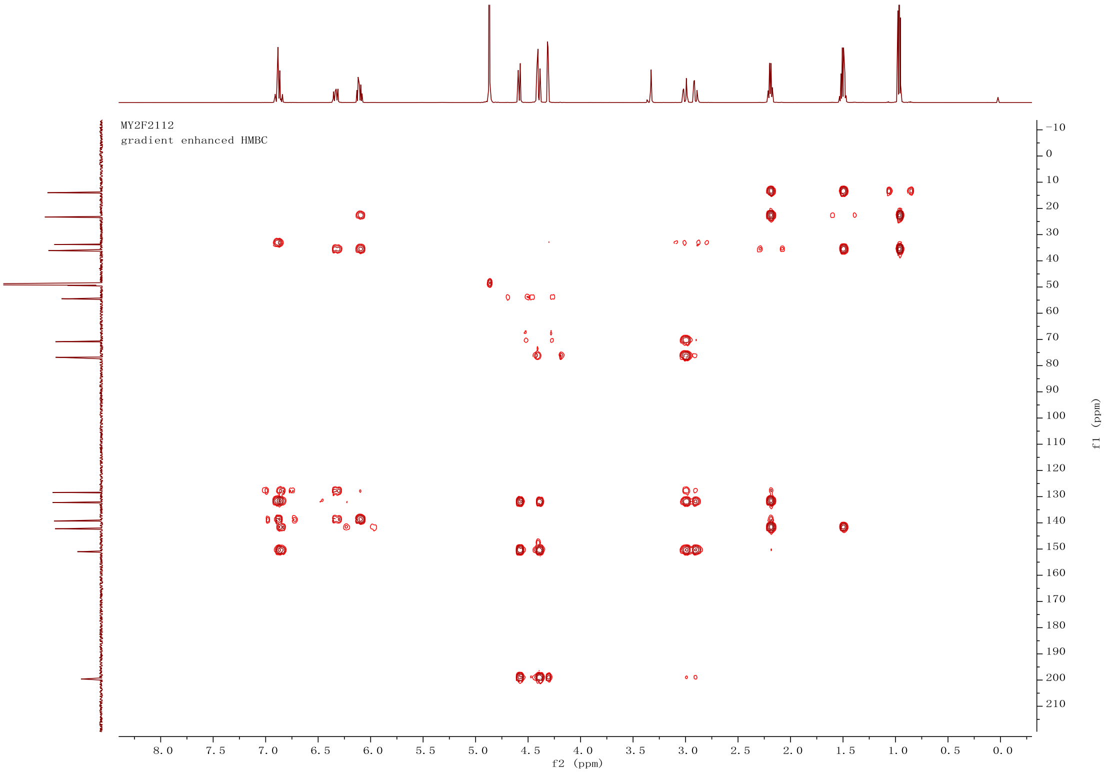

Figure S23 The HMBC (MeOH-*d*$_4$) spectrum of palitantin B (**7**)

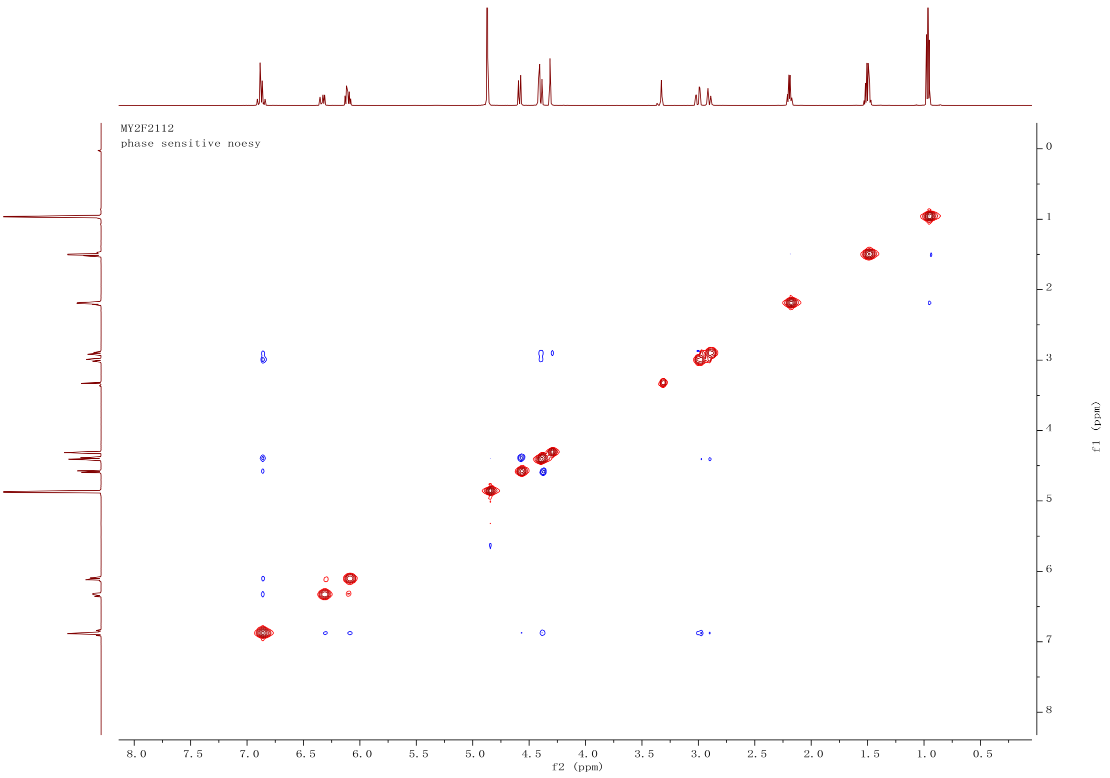

Figure S24 The NOSEY (MeOH-d_4) spectrum of palitantin B (**7**)

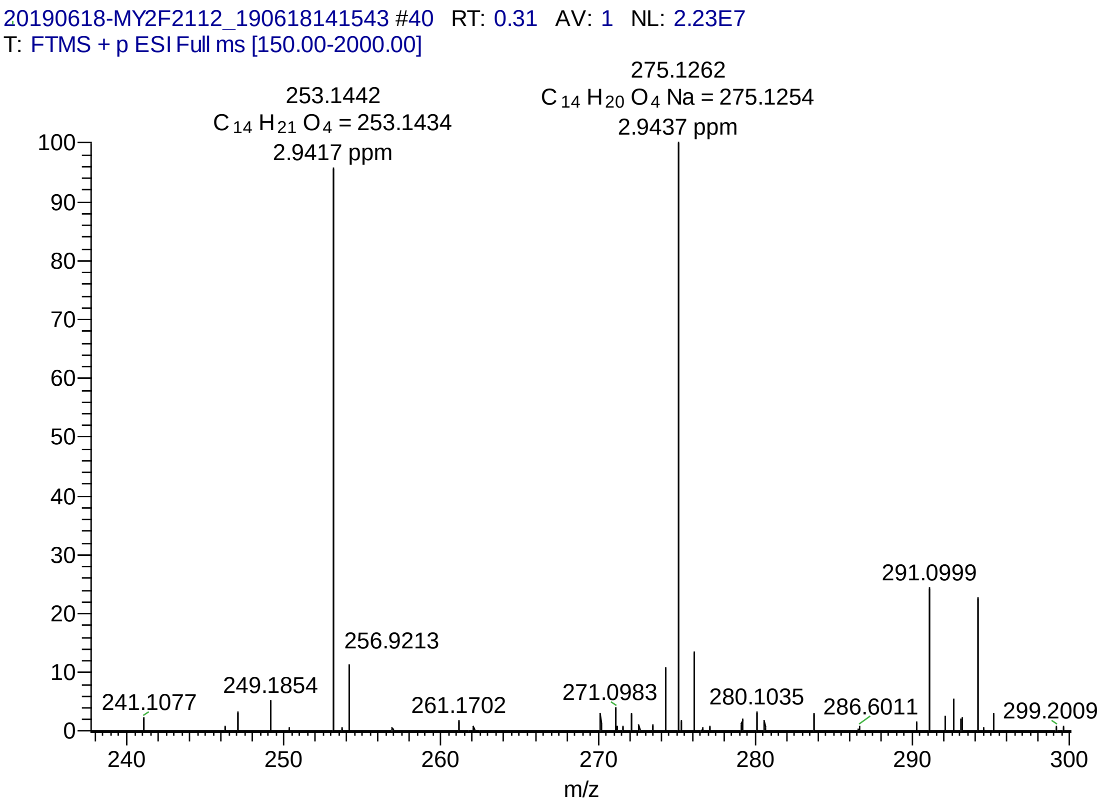

Figure S25 The HRESIMS spectrum of palitantin B (**7**)

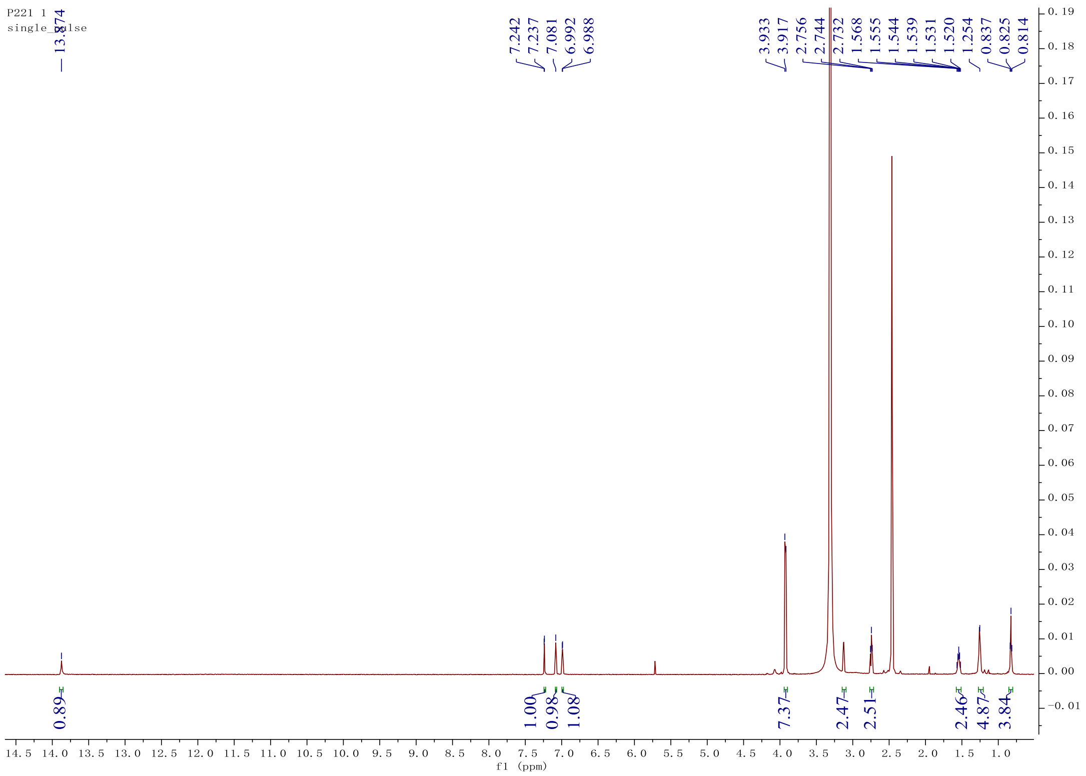

Figure S26 The ^{1}H NMR (600 MHz, DMSO-d_6) spectrum of 1,3-di-*O*-methyl-norsolorinic acid (**8**)

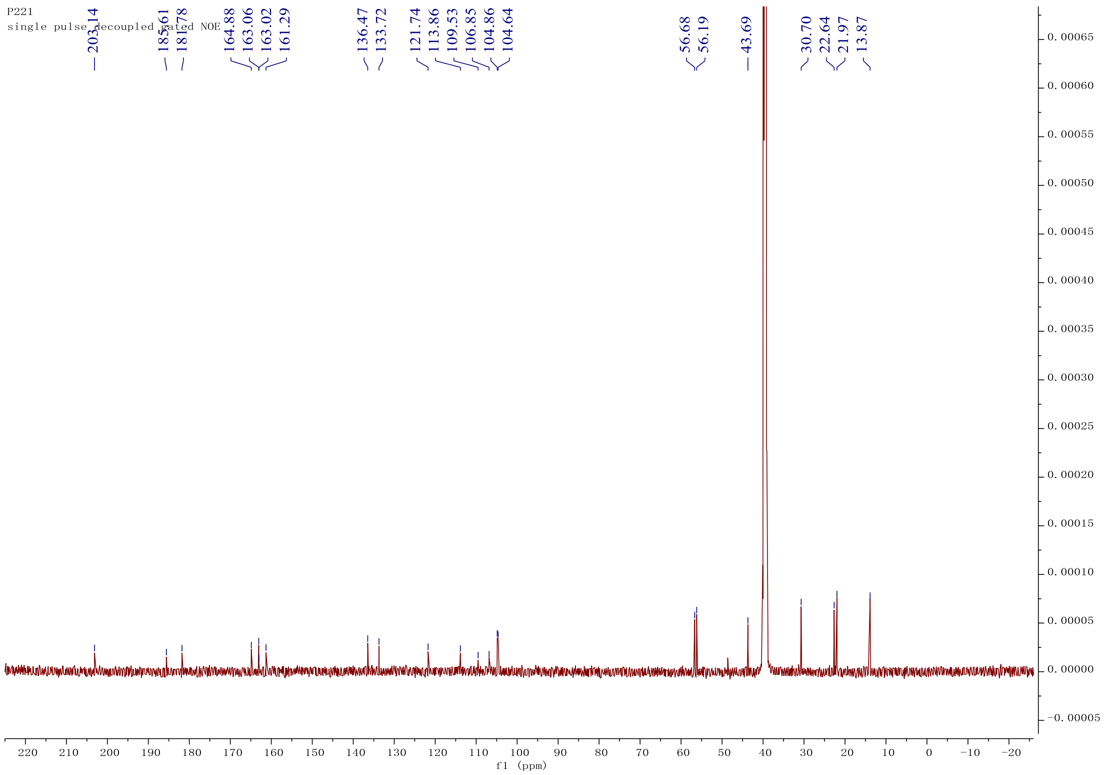

Figure S27 The ^{13}C NMR (150 MHz, DMSO-d_6) spectrum of 1,3-di-*O*-methyl-norsolorinic acid (**8**)

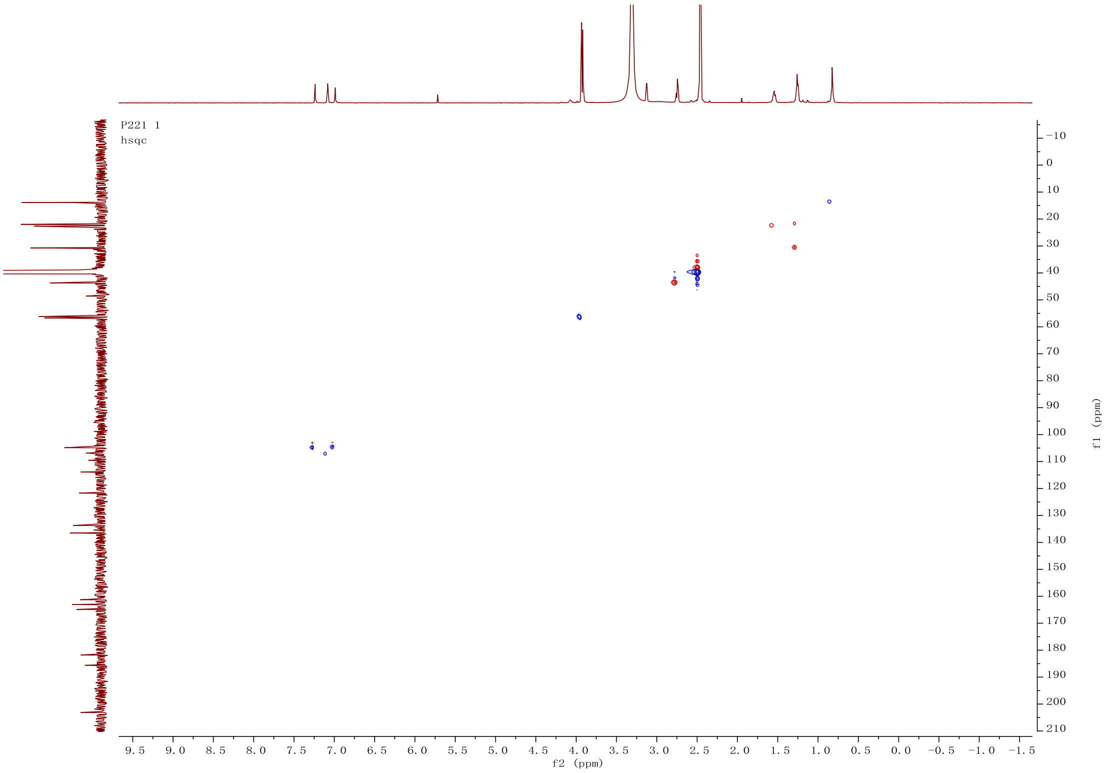

Figure S28 The HSQC (DMSO-d_6) spectrum of 1,3-di-*O*-methyl-norsolorinic acid (**8**)

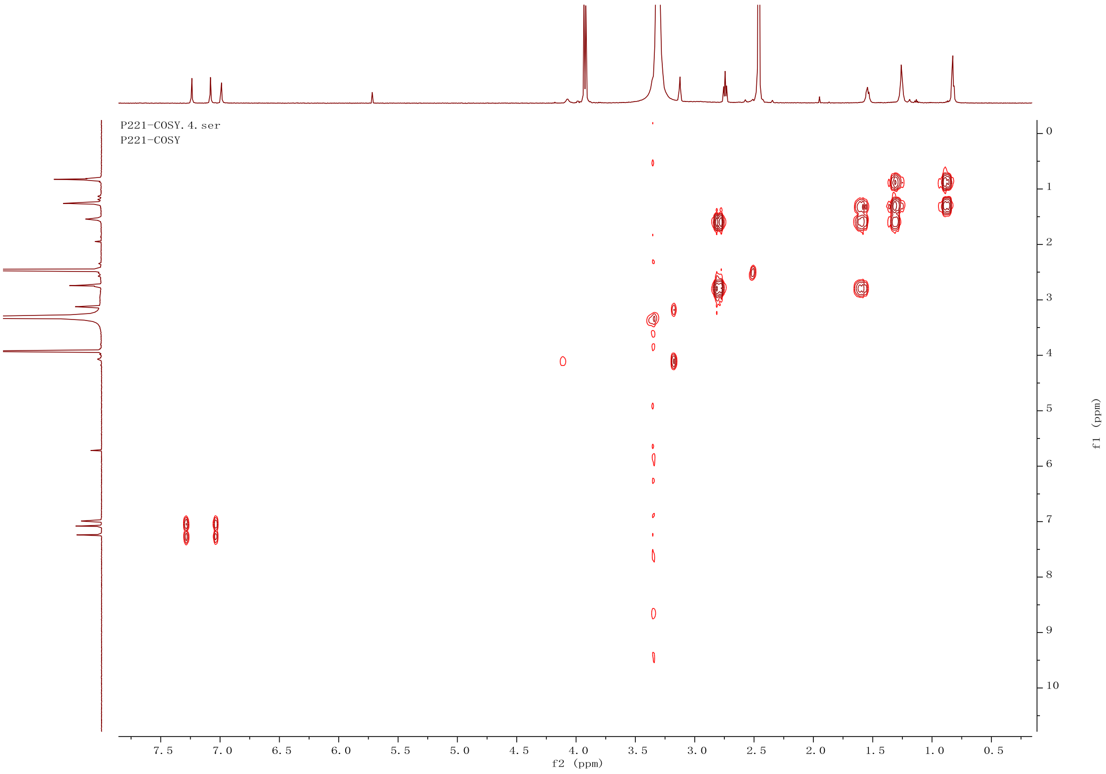

Figure S29 The ^1H-^1H COSY ($CDCl_3$) spectrum of 1,3-di-*O*-methyl-norsolorinic acid (**8**)

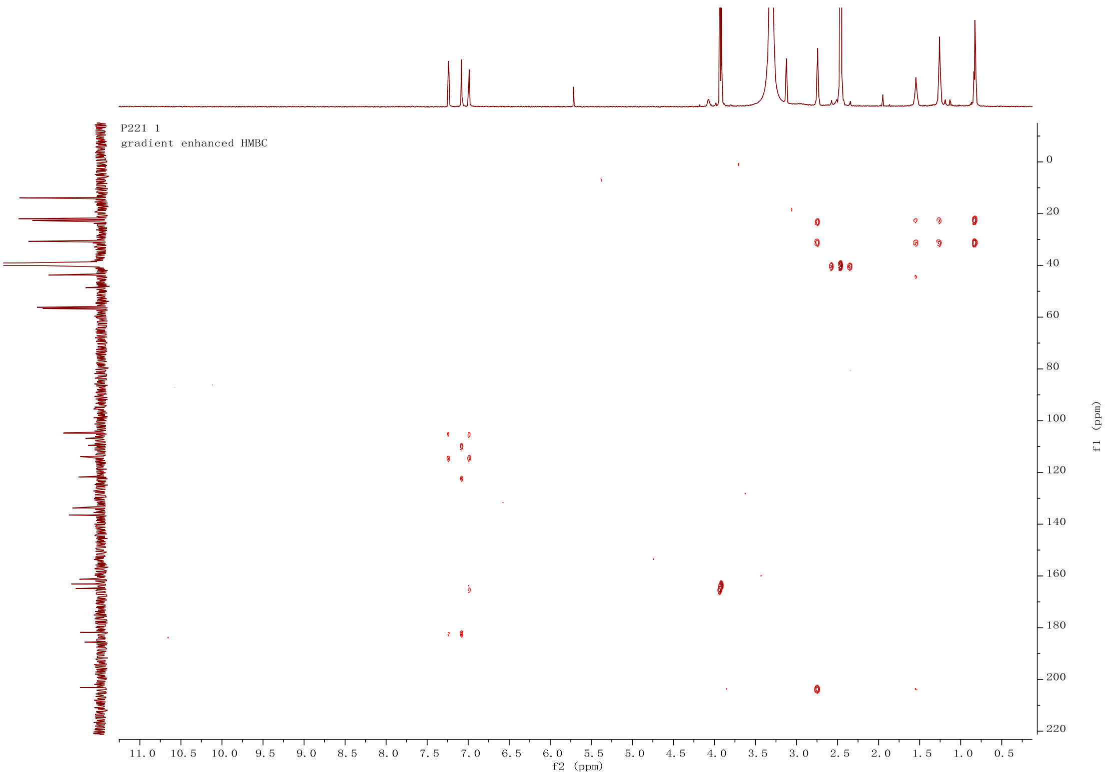

Figure S30 The HMBC ($CDCl_3$) spectrum of 1,3-di-*O*-methyl-norsolorinic acid (**8**)

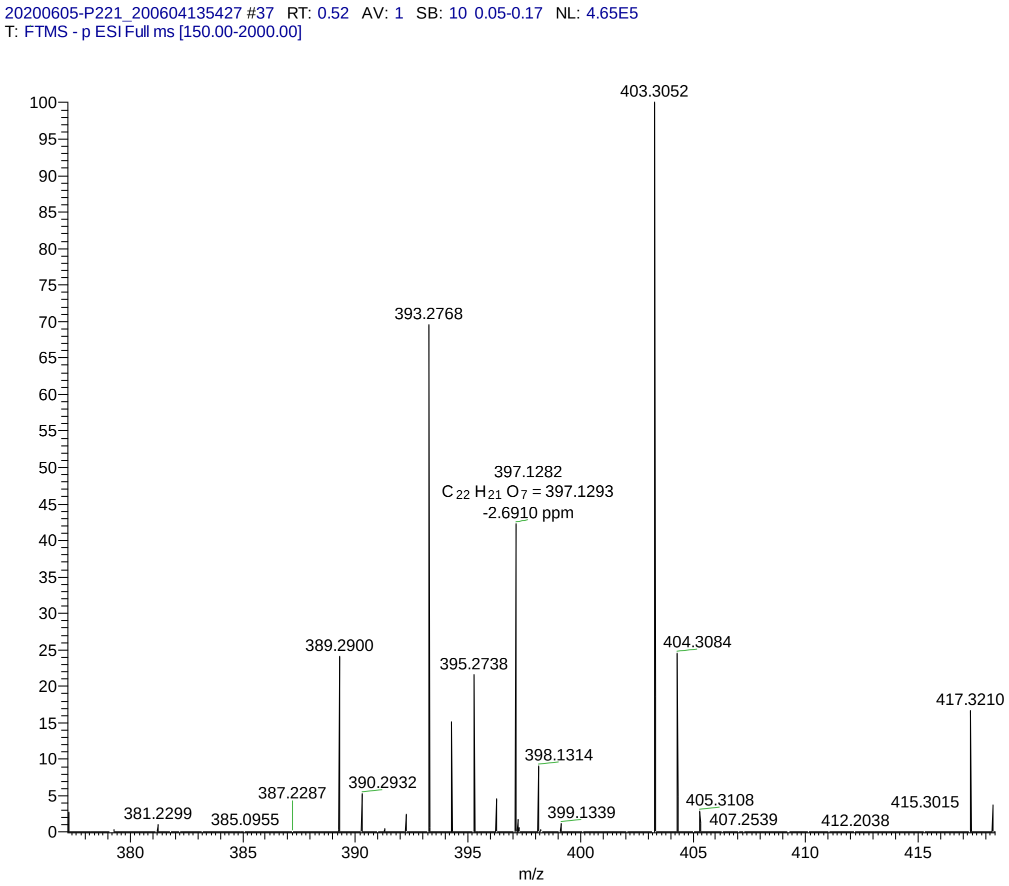

Figure S31 The HRESIMS spectrum of 1,3-di-*O*-methyl-norsolorinic acid (**8**)

Figure S32 The lowest-energy conformer (1*S*,3*S*,8*R*,9*S*,10*S*)-**1** in ECD calculation

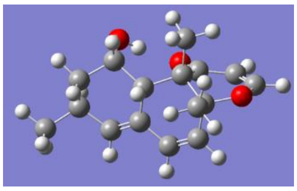

E = - 847.6306466 a.u.

Standard orientation:

Center Number	Atomic Number	Atomic Type	Coordinates (Angstroms) X	Y	Z
1	6	0	-2.945423	0.407753	0.996237
2	6	0	-3.316537	-0.169762	-0.376949
3	6	0	-2.199505	-1.034165	-0.895668
4	6	0	-0.933436	-0.978104	-0.449143
5	6	0	-0.496637	-0.020800	0.648234
6	6	0	-1.556762	1.062684	0.988614
7	6	0	0.092622	-1.895301	-0.929243
8	6	0	1.358853	-1.879257	-0.485797
9	6	0	1.864892	-0.874198	0.525892
10	6	0	0.992413	0.426250	0.439365
11	8	0	3.278856	-0.587849	0.193519
12	6	0	3.522192	0.202553	-0.851486
13	6	0	2.598231	0.956038	-1.476174
14	6	0	1.244614	1.049407	-0.961802
15	8	0	0.379472	1.670469	-1.570687
16	6	0	1.407483	1.480067	1.486235
17	6	0	1.994394	-1.501142	1.916741
18	6	0	-4.648000	-0.933004	-0.336469
19	8	0	-1.593278	2.209270	0.156233
20	1	0	-3.678028	1.162453	1.296760
21	1	0	-2.974791	-0.389811	1.750955
22	1	0	-3.438447	0.675057	-1.069982
23	1	0	-2.446191	-1.753875	-1.675863
24	1	0	-0.468131	-0.616136	1.569586
25	1	0	-1.343255	1.418496	2.004545
26	1	0	-0.207105	-2.646175	-1.656532
27	1	0	2.081113	-2.614386	-0.828752
28	1	0	4.566584	0.169143	-1.146788
29	1	0	2.857782	1.539202	-2.350534
30	1	0	2.479192	1.685153	1.444363
31	1	0	0.873558	2.416257	1.317843
32	1	0	1.170419	1.140100	2.497201

33	1	0	2.635851	-2.382072	1.847703
34	1	0	2.452986	-0.806773	2.622741
35	1	0	1.030173	-1.825274	2.306315
36	1	0	-4.593364	-1.793107	0.338948
37	1	0	-5.458137	-0.285791	0.011540
38	1	0	-4.925197	-1.305357	-1.327560
39	1	0	-1.011778	2.083664	-0.616534

Figure S33 The lowest-energy conformer (1*S*,3*S*,8*R*,9*S*,10*S*)-**2** in ECD calculation

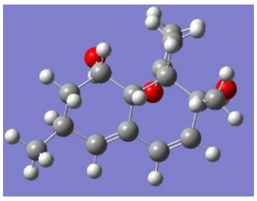

E = -810.7333125 a.u.

Standard orientation:

Center Number	Atomic Number	Atomic Type	Coordinates (Angstroms) X	Y	Z
1	6	0	-2.730942	0.683238	1.007225
2	6	0	-3.231983	-0.054978	-0.242248
3	6	0	-2.199092	-1.037085	-0.727525
4	6	0	-0.907819	-1.051893	-0.352425
5	6	0	-0.342457	-0.077461	0.669698
6	6	0	-1.273703	1.136839	0.882953
7	6	0	0.010893	-2.062164	-0.855252
8	6	0	1.318612	-2.054670	-0.578027
9	6	0	2.020991	-1.015528	0.260691
10	6	0	1.156966	0.310561	0.422623
11	6	0	1.314236	1.103513	-0.909970
12	6	0	2.166837	2.355135	-0.941402
13	8	0	0.798412	0.696334	-1.933459
14	6	0	2.421896	-1.644319	1.602077
15	8	0	3.286099	-0.686897	-0.365264
16	6	0	-4.586809	-0.734552	0.007524
17	8	0	-1.112769	2.142858	-0.122222
18	6	0	1.669379	1.139756	1.614164
19	1	0	-0.338587	-0.613889	1.629320
20	1	0	-3.358839	1.555603	1.212035
21	1	0	-2.814208	0.017686	1.876146
22	1	0	-3.397061	0.688036	-1.039294
23	1	0	-2.535692	-1.782129	-1.447733

24	1	0	-0.991750	1.646158	1.806511
25	1	0	-0.402415	-2.848747	-1.482048
26	1	0	1.965633	-2.844416	-0.953720
27	1	0	1.706189	3.149183	-0.349424
28	1	0	3.159835	2.165244	-0.527957
29	1	0	2.255598	2.691442	-1.974048
30	1	0	1.552096	-1.842488	2.231215
31	1	0	2.924184	-2.594766	1.411533
32	1	0	3.118043	-1.004544	2.145940
33	1	0	3.158942	-0.655595	-1.322700
34	1	0	-4.505058	-1.502189	0.783424
35	1	0	-5.336467	-0.006681	0.331786
36	1	0	-4.963060	-1.216645	-0.899587
37	1	0	-1.212588	1.746490	-0.998103
38	1	0	1.219878	2.132585	1.633431
39	1	0	1.427320	0.655510	2.562347
40	1	0	2.752044	1.267440	1.577844

Figure S34 The lowest-energy conformer (2*R*,3*R*)-**7** in ECD calculation

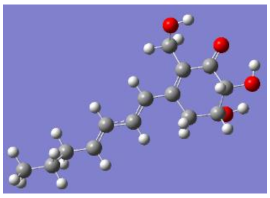

E = - 846.6613052 a.u.

Standard orientation:

Center Number	Atomic Number	Atomic Type	Coordinates (Angstroms) X	Y	Z
1	6	0	-1.953285	0.910922	0.107299
2	6	0	-0.887535	0.052132	0.031544
3	6	0	-1.085767	-1.417172	-0.301305
4	6	0	-2.508109	-1.944378	-0.159956
5	6	0	-3.495063	-0.960030	-0.770586
6	6	0	-3.304642	0.430308	-0.174657
7	6	0	-1.899263	2.364740	0.536386
8	8	0	-2.468855	3.251310	-0.420516
9	6	0	0.475999	0.504374	0.234731
10	6	0	1.590026	-0.267140	0.205425
11	6	0	2.927484	0.251179	0.377494
12	6	0	4.035985	-0.510594	0.362654
13	6	0	5.436327	-0.010933	0.534594
14	6	0	6.344596	-0.323761	-0.670365

15	6	0	7.785093	0.147488	-0.463212
16	8	0	-2.786788	-2.130033	1.221606
17	8	0	-4.813562	-1.416062	-0.551324
18	8	0	-4.307138	1.121117	0.010799
19	1	0	-0.739903	-1.594259	-1.327689
20	1	0	-0.450825	-2.029359	0.342988
21	1	0	-2.591888	-2.905862	-0.684309
22	1	0	-3.294103	-0.879666	-1.852269
23	1	0	-2.421092	2.460887	1.497708
24	1	0	-0.881268	2.712535	0.689400
25	1	0	-3.418440	3.071493	-0.444952
26	1	0	0.625061	1.566280	0.396045
27	1	0	1.513049	-1.339430	0.045859
28	1	0	3.025293	1.325401	0.531639
29	1	0	3.925975	-1.584698	0.206434
30	1	0	5.427907	1.068516	0.722562
31	1	0	5.874974	-0.476811	1.428702
32	1	0	6.334105	-1.403949	-0.859530
33	1	0	5.923732	0.145386	-1.566570
34	1	0	8.407868	-0.086727	-1.330815
35	1	0	7.833100	1.229648	-0.306575
36	1	0	8.240752	-0.331501	0.409205
37	1	0	-3.737488	-2.293602	1.301322
38	1	0	-5.357149	-0.627757	-0.383950

Figure S35 The lowest-energy conformer (1*S*,3*S*,8*R*,9*R*,10*S*)-**6** in ECD calculation

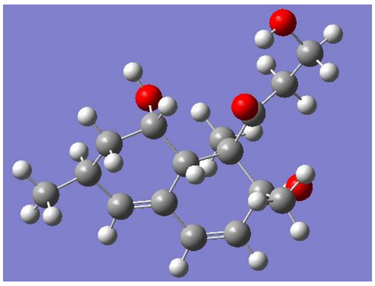

E = -925.3011515 a.u.

Standard orientation:

Center Number	Atomic Number	Atomic Type	Coordinates (Angstroms) X	Y	Z
1	6	0	-2.574559	-1.700422	-0.653732
2	6	0	-3.575922	-0.896919	0.186261

3	6	0	-3.133809	0.538405	0.264897
4	6	0	-1.879160	0.959723	0.031168
5	6	0	-0.746728	0.019316	-0.350389
6	6	0	-1.125323	-1.472241	-0.213512
7	6	0	-1.541932	2.377252	0.055068
8	6	0	-0.289172	2.827024	-0.091389
9	6	0	0.932466	1.947787	-0.235486
10	6	0	0.603156	0.475751	0.311636
11	6	0	1.743802	-0.454461	-0.144520
12	6	0	2.991052	-0.580301	0.712154
13	6	0	4.127892	-1.329816	0.018408
14	8	0	3.807300	-2.681786	-0.279170
15	8	0	1.664028	-1.061210	-1.200824
16	8	0	-0.932669	-1.921161	1.140028
17	6	0	0.502199	0.525351	1.853396
18	8	0	2.010727	2.534707	0.521794
19	6	0	-5.005770	-1.021523	-0.357862
20	6	0	1.442438	1.990624	-1.682166
21	1	0	-0.598013	0.142024	-1.429099
22	1	0	-2.806480	-2.771615	-0.599917
23	1	0	-2.671182	-1.417743	-1.709836
24	1	0	-3.570566	-1.309061	1.205921
25	1	0	-3.893848	1.278328	0.514420
26	1	0	-0.458768	-2.041662	-0.865233
27	1	0	-2.357449	3.086232	0.178499
28	1	0	-0.083565	3.894954	-0.113970
29	1	0	3.313755	0.425169	1.004365
30	1	0	2.717349	-1.096579	1.640052
31	1	0	4.426242	-0.795368	-0.893165
32	1	0	4.997535	-1.363686	0.677841

33	1	0	3.096226	-2.657838	-0.933869
34	1	0	-1.015114	-2.882055	1.157248
35	1	0	0.255719	-0.454537	2.251965
36	1	0	1.435297	0.854811	2.310764
37	1	0	-0.285869	1.212028	2.166223
38	1	0	1.672186	2.815941	1.380676
39	1	0	-5.718524	-0.475819	0.267778
40	1	0	-5.080612	-0.620892	-1.373911
41	1	0	-5.325738	-2.067450	-0.386694
42	1	0	2.434736	1.542564	-1.758833
43	1	0	0.776140	1.472022	-2.370826
44	1	0	1.521681	3.032534	-1.998617

Table S1 The antimicrobial activities of isolated compounds **14**–**15** (MIC, μg/mL)

Strains	**14**	**15**	Control
S. aureus[a]	3.125	12.5	3.125
A. salmonicida[b]	3.125	>25	0.39
P. angustum[b]	>25	25	1.56

[a, b] Ciprofloxacin, sea-nine 211 and were used as the positive controls, respectively

Table S2 The antioxidant activities of compound **15**

Antioxidant assays	**15**	ascorbic acid[a]
DPPH radicals scavenging	IC_{50} = 34.1 μM	IC_{50} = 115.1 μM
Reduction of Fe^{3+}	FRAP = 9.0 mM	FRAP = 5.6 mM

[a] Ascorbic acid was used as the positive control
